# The FABP12/PPARγ pathway promotes metastatic transformation by inducing epithelial‐to‐mesenchymal transition and lipid‐derived energy production in prostate cancer cells

**DOI:** 10.1002/1878-0261.12818

**Published:** 2020-10-23

**Authors:** Rong‐Zong Liu, Won‐Shik Choi, Saket Jain, Deepak Dinakaran, Xia Xu, Woo Hyun Han, Xiao‐Hong Yang, Darryl D. Glubrecht, Ronald B. Moore, Hélène Lemieux, Roseline Godbout

**Affiliations:** ^1^ Department of Oncology Cross Cancer Institute University of Alberta Edmonton AB Canada; ^2^ Faculty Saint‐Jean University of Alberta Edmonton AB Canada; ^3^ Department of Surgery University of Alberta Edmonton AB Canada

**Keywords:** bioenergetics, epithelial–mesenchymal transition, fatty acid‐binding proteins, metastasis, peroxisome proliferator‐activated receptor gamma, prostate cancer

## Abstract

Early stage localized prostate cancer (PCa) has an excellent prognosis; however, patient survival drops dramatically when PCa metastasizes. The molecular mechanisms underlying PCa metastasis are complex and remain unclear. Here, we examine the role of a new member of the fatty acid‐binding protein (FABP) family, FABP12, in PCa progression. *FABP12* is preferentially amplified and/or overexpressed in metastatic compared to primary tumors from both PCa patients and xenograft animal models. We show that FABP12 concurrently triggers metastatic phenotypes (induced epithelial‐to‐mesenchymal transition (EMT) leading to increased cell motility and invasion) and lipid bioenergetics (increased fatty acid uptake and accumulation, increased ATP production from fatty acid β‐oxidation) in PCa cells, supporting increased reliance on fatty acids for energy production. Mechanistically, we show that FABP12 is a driver of PPARγ activation which, in turn, regulates FABP12's role in lipid metabolism and PCa progression. Our results point to a novel role for a FABP‐PPAR pathway in promoting PCa metastasis through induction of EMT and lipid bioenergetics.

## Graphical abstract

FABP12 promotes fatty acid (FA) uptake and facilitates FA trafficking to lipid droplets (storage), mitochondria (beta‐oxidation), and the nucleus (PPARγ activation). In turn, FABP12‐induced PPARγ activation regulates expression of mitochondrial FA metabolic enzymes (for energy production) and Slug (for EMT), thereby inducing metastatic transformation.

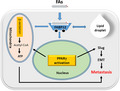

**Abbreviations**
AR: androgen receptor
ATP: adenosine triphosphate
CN: copy number
CPT1: carnitine palmitoyltransferase I
CS: citrate synthase
EMT: epithelial–mesenchymal transition
ET: electron transfer‐state
FABP: fatty acid‐binding protein
LD: lipid droplet
OA: oleic acid
PCa: prostate cancer
PPAR: peroxisome proliferator‐activated receptor
PPRE: peroxisome proliferator‐activated receptor response element
TZD: thiazolidinediones

## 1 Introduction

Prostate cancer (PCa) is the most prevalent cancer in men. While localized PCa can usually be cured, metastatic disease is often resistant to treatment, with eventual relapse and death [1]. It is therefore imperative that we understand the underlying causes of PCa metastasis and identify the key molecules that drive PCa metastasis. Three features limit the clinical management of PCa: cancer heterogeneity, lack of molecular signatures to stratify tumor subtypes [2], and reliance of PCa cells on fatty acid oxidation for their energy supply rather than glucose consumption [3].

Lipid metabolic reprogramming is regarded as a hallmark of cancer progression, and it is broadly accepted that cancer cells have a markedly increased need for lipids [4, 5]. This is particularly true for PCa [6, 7, 8, 9]. Epidemiological studies reveal a positive relationship between the consumption of dietary fats, lipid metabolism, and development of PCa [7, 10, 11]. It is believed that aberrant metabolic adaptations, such as enhanced aerobic glycolysis and increased lipid utilization, are crucial for cancer cells to separate from the primary tumor mass, invade the surrounding stroma, overcome nutrient and energy deficits, and metastasize to secondary sites [12]. Recent studies suggest emerging roles for dysregulated lipid accumulation and metabolism in metastatic cancers [13, 14], especially in PCa [15, 16]. Lipids or fatty acids not only provide energy for cancer cell growth and dissemination [3, 17, 18], but also serve as cell membrane components that exert profound effects on signal transduction and cell growth properties [19]. Furthermore, fatty acids function as activating ligands for nuclear receptors such as peroxisome proliferator‐activated receptors (PPARs), which regulate gene pathways implicated in PCa lipid homeostasis, tumorigenesis, and cancer progression [19, 20, 21].

Because of their hydrophobic nature, fatty acids require chaperones such as fatty acid‐binding proteins (FABPs) for their transport within the cell [22]. FABPs are important determinants of intracellular accumulation, distribution, utilization, and fate of fatty acids [23, 24]. There are ten *FABP* genes in mammals, with each *FABP* displaying distinct tissue distribution patterns and ligand preference [22, 25]. FABPs are receiving increasing attention in the field of oncology because of their demonstrated roles in cancer progression, and proposed roles in the prevention and treatment of cancer [26, 27, 28, 29], particularly as related to PPAR function [30, 31]. While a number of studies point to FABP involvement in PCa metastasis [32, 33, 34], the precise mechanism underlying FABP action remains elusive. Particularly, the roles of FABPs in epithelial‐to‐mesenchymal transition (EMT), a priming process leading to metastasis of epithelium‐derived carcinomas [35], and dysregulation of energy production, a critical event promoting metastasis in various cancer types [13, 14], have yet to be determined in PCa.

The *FABP12* gene is located on chromosome at 8q21.13, a frequently amplified region in human PCa [36, 37, 38]. Genes previously reported to be amplified and/or overexpressed in PCa include Tumor Protein D52 (*TPD52*, 8q21.13) [37, 39], Elongin C (*ELOC*, 8q21.11) [36], ZBTB10 (8q21.13) [39], and FABP5 (8q21.13) [40, 41]. Here, we show that FABP12, the newest member of the FABP family which is also located at 8q21.13 [25], is preferentially amplified and overexpressed in metastatic PCa, drives migration and invasion in PCa cells through activation of PPARγ which, in turn, induces EMT and increased reliance on fatty acids for ATP production.

## 2 Materials and methods

### 2.1 Cell lines, inhibitors, and transfections

The PC3 and DU145 cell lines were obtained from ATCC (Manassas, VA, USA) and frozen in liquid nitrogen within two passages of receipt. Cells were cultured in DMEM supplemented with 10% FBS and penicillin/streptomycin. GW9662, CAS300657‐03‐8 (T4B), and etomoxir (Sigma‐Aldrich, St. Louis, MO, USA) were used to block the activity of PPARγ, FABP12, and CPT1, respectively. For stable transfections, PC3 cells were transfected with pREP4 or pREP4‐FABP12 using polyethylenimine (PEI), followed by selection in hygromycin. Three stable clonal populations of PC3‐pREP4 cells and four stable clonal populations of PC3‐pREP4‐FABP12 cells were selected for further analysis. For transient transfections, DU145 or PC3 cells were transfected with either pcDNA3.1_HA or pcDNA3.1‐HA‐FABP12 using jetPRIME (Polyplus Transfection, New York, NY, USA). For PPARγ knockdown, control and PC3‐FABP12 cells were transfected with 10 nm scrambled (control) or PPARγ‐specific siRNAs (Thermo Fisher; sequences listed in Table S1) using the RNAiMAX transfection reagent (Thermo Fisher, Waltham, MA, USA).

### 2.2 PCa xenograft mouse model and immunohistochemistry

Three million cells in 100 μL PBS were injected into the right flank of NSG mice. Mice were euthanized 8 weeks postinjection. Ipsilateral and contralateral axillary lymph nodes, and sites of metastasis previously reported for PC3 cells [42] were examined. Tumors were removed and fixed in 4% paraformaldehyde containing 10% sucrose for 48 h at 4 °C. Tissue sections were processed as previously described [43] and immunostained with anti‐FABP12 antibody (Customized Ab by BIOMATIK, Kitchener, Ontario, Canada; 1 : 300 at 0.79 μg·mL^−1^), followed by EnVision + anti‐rabbit HRP‐labeled polymer (DakoCytomation, Carpinteria, CA, USA). Signal specificity was determined using FABP12 peptide (antigen for FABP12 antibody production, 13 μg·mL^−1^) as a competitor. Tissues were counterstained with hematoxylin.

Animal ethics approval was obtained from the Cross Cancer Institute Animal Care Committee—protocol AC17233. All methods used for monitoring tumor growth, euthanasia, and collection of tumor tissue were carried out in accordance with the Canadian Council on Animal Care (CCAC) Guidelines and Policies. Four to five mice were used for each experiment, with experiments carried out four times.

### 2.3 Cell motility, migration, and invasion assays

The scratch (wound healing) assay was used to measure cell motility [44]. Cells transiently (DU145) or stably (PC3) transfected with FABP12 expression constructs, or their corresponding empty vectors, were seeded in triplicate in 12‐well plates. A scratch was made across the monolayer of cells with a P20 pipette tip when cells reached confluency. Cells were cultured for an additional 36 h (DU145) or 30 h (PC3). Dynamic images (15‐minute intervals) at two separate positions in each well were captured using digital phase‐contrast microscopy (Axiovert 200M, Zeiss). The percentage open area of the scratch at different time points was analyzed with TScratch software [45]. The open area at each time point is represented relative to the 0‐h time point which was set at 100%. Cell migration and invasion properties were analyzed using Transwell inserts (8 μm pore size) and BioCoat Matrigel Invasion Chambers, respectively (BD Biosciences, San Jose, CA, USA). Fifty thousand cells in serum‐free medium were seeded in the upper chambers of the inserts. Cells were allowed to migrate through the Transwell or Matrigel membranes toward the lower chamber containing DMEM supplemented with 10% FBS for 48–72 h (Transwell) or 72 h (Matrigel). Cells were then fixed, stained with crystal violet, and counted.

### 2.4 Immunofluorescence analysis

Cells were cultured on coverslips for 48 h, fixed in 4% paraformaldehyde for 10 min, and permeabilized in 0.25% Triton X‐100 for 10 min. Cells were immunostained with anti‐Slug (1 : 200; Cell Signaling, Danvers, MA, USA) or anti‐E‐cadherin (1 : 200; Cell signaling) antibodies for 1 h at room temperature, followed by incubation in Alexa 555‐conjugated donkey anti‐rabbit (Life Technologies, Thermo Fisher) for 1 h at room temperature. Coverslips were mounted with Mowiol 4‐88 (Sigma‐Aldrich) mounting medium containing DAPI and images acquired using a Zeiss LSM510 confocal microscope (Oberkochen, Germany).

### 2.5 Gel shift assays

Gel shifts were carried out using a peroxisome proliferator‐activated receptor response element (PPRE) probe (Table S1) as previously described [43] Briefly, annealed oligonucleotides were radiolabeled with [α‐^32^P]dCTP. Nuclear protein extracts (1 μg) were pre‐incubated with 1 μg poly(dIdC) in binding buffer, followed by addition of labeled probe. For supershift assays, 1 μL of anti‐PPARα, anti‐PPARβ/δ, or anti‐PPARγ antibody was added to the binding reaction 10 min after addition of the labeled probe and incubated at room temperature for 30 min. DNA–protein complexes were resolved in a 5% native polyacrylamide gels in 0.5× Tris/borate/EDTA buffer for 1.5 h. The gels were vacuum‐dried and exposed to X‐ray film.

### 2.6 Luciferase reporter assay

Cells were seeded in 12‐well plates (30 000 cells/well) and transfected with the luciferase reporter construct (PPRE 3‐TK‐Luc; Addgene, Waterton, MA, USA) using polyethyleneimine (PEI). Transfected cells were then harvested and protein lysates prepared using the luciferase cell culture lysis reagent (CCLR; Promega). Luciferase activity was measured using the Luciferase Assay System (Promega, Madison, WI, USA) and quantified with the FLUOstar OPTIMA microplate reader (BMG Labtech, Ortenberg, Germany) following the manufacturer's instructions.

### 2.7 Lipid droplet analysis

Cells were cultured on coverslips for 2 days, fixed with 4% paraformaldehyde, and stained with Nile Red (0.5 μg·mL^−1^) for 15 min. To measure fatty acid‐induced lipid droplet formation, we used the Lipid Droplet Fluorescence Assay Kit (Cayman Chemical, Ann Harbor, MI, USA). Briefly, cells were seeded in 96‐well plate at 15 000 cells/well and treated with oleic acid at increasing concentrations (0, 125 μg·mL^−1^, 500 μg·mL^−1^) for 24 h. Cells were then stained with Nile Red, and fluorescence intensity was measured using a FLUOstar Omega microplate reader (BMG Labtech).

### 2.8 High‐resolution respirometry analysis of mitochondrial function

Cell respiration was measured at 37 °C using an Oxygraph‐2k (Oroboros Instruments, Innsbruck, Austria). Cells (in 2 mL culture medium) were added to each chamber of the Oxygraph and the O_2_ flow (pmol·min^−1^·million^−1^ cells) measured under routine condition (ROUTINE state), resting state (LEAK, after inhibition of ATP synthase with 2 μg·mL^−1^ of oligomycin), electron transfer capacity (ET state; maximal O_2_ consumption after stepwise titration to an optimal concentration of the protonophore carbonyl cyanide *p*‐[trifluoromethoxy] phenylhydrazone, FCCP), and residual oxygen consumption (ROX state, after inhibition of complex III with 2.5 μm antimycin A). ATP‐linked respiration is defined as ROUTINE with subtraction of LEAK. For detection of fatty acid‐related respiration, cells were treated with 100 μm of etomoxir (Sigma) [46], a CPT1 inhibitor, for 24 h prior to mitochondrial respirometry analysis.

### 2.9 Citrate synthase activity analysis

Stable control and FABP12‐expressing PC3 cells were harvested and washed twice with ice‐cold PBS, resuspended in ice‐cold assay buffer (0.05 m Tris/HCl, 1.3 mm MgCl_2_, pH = 8.0), homogenized with a Dounce homogenizer, and centrifuged 5 min at 13 500 ***g*** at 4 °C. The supernatant (50 and 100 μL) was mixed with 0.1 mm DTNB [5,5′‐dithiobis(2‐nitrobenzoic acid)], 0.25% Triton X‐100, 0.5 mm oxaloacetate, and 0.31 mm acetyl‐CoA, and citrate synthase (CS) activity was measured using a Ultraspec 2100 Pro Spectrophotometer (GE Healthcare, Chicago, IL, USA) to measure OD_412_. Results were acquired and analyzed using SWIFT II reaction kinetics software (Biochrom, Holliston, MA, USA).

### 2.10 Measurement of cellular ATP levels

Cells were grown in 12‐well plates (30 000 cells/well) for three days. Cells were then trypsinized, resuspended in DMEM, and counted. ATP levels of intact cells (in triplicate) were measured using the ATP Determination Kit (Invitrogen, Thermo Fisher) following the manufacturer's instructions. ATP levels for each sample were normalized to cell numbers.

### 2.11 RT‐PCR and western blot analysis

Total RNA from PCa cell lines was prepared using the TRIzol reagent (Invitrogen). First‐strand cDNA was synthesized using SuperScript reverse transcriptase II (Invitrogen). PCR amplification was performed with gene‐specific primers (Table S1) as previously described [25].

Cell protein lysates were prepared as previously described [47]. Whole‐cell (40 μg per lane), cytoplasmic (40 μg per lane), or nuclear protein extracts (20 μg per lane) were separated by SDS/PAGE and transferred to nitrocellulose membranes. Membranes were immunoblotted with primary and secondary antibodies (listed in Table S2).

### 2.12 Databases and statistical analysis

Gene copy numbers and mRNA levels in human PCa tissues were measured using different PCa cohorts. Clinical data from the MSKCC dataset [48] were used for patient survival analysis. All datasets were accessed through cBioportal (www.cbioportal.org/datasets). Statistical analyses were performed using medcalc version 14.12.0 (MedCalc Software, Ostend, Belgium). Log‐rank test was used to compare Kaplan–Meier survival probabilities between patient populations stratified based on mRNA levels (FABP12, PPARγ) or amplification status (FABP12). One‐way ANOVA (when comparing more than two groups) or two‐sided Student's *t*‐test (when comparing two groups) was employed to compute the significance of the difference between patient groups or experimental treatments. Two‐way ANOVA was used to examine the significance of effects from two independent experimental factors (i.e., scratch assay, Nile Red assay).

## 3 Results

### 3.1 *FABP12* is preferentially amplified and overexpressed in metastatic PCa

**Fig. 1:**
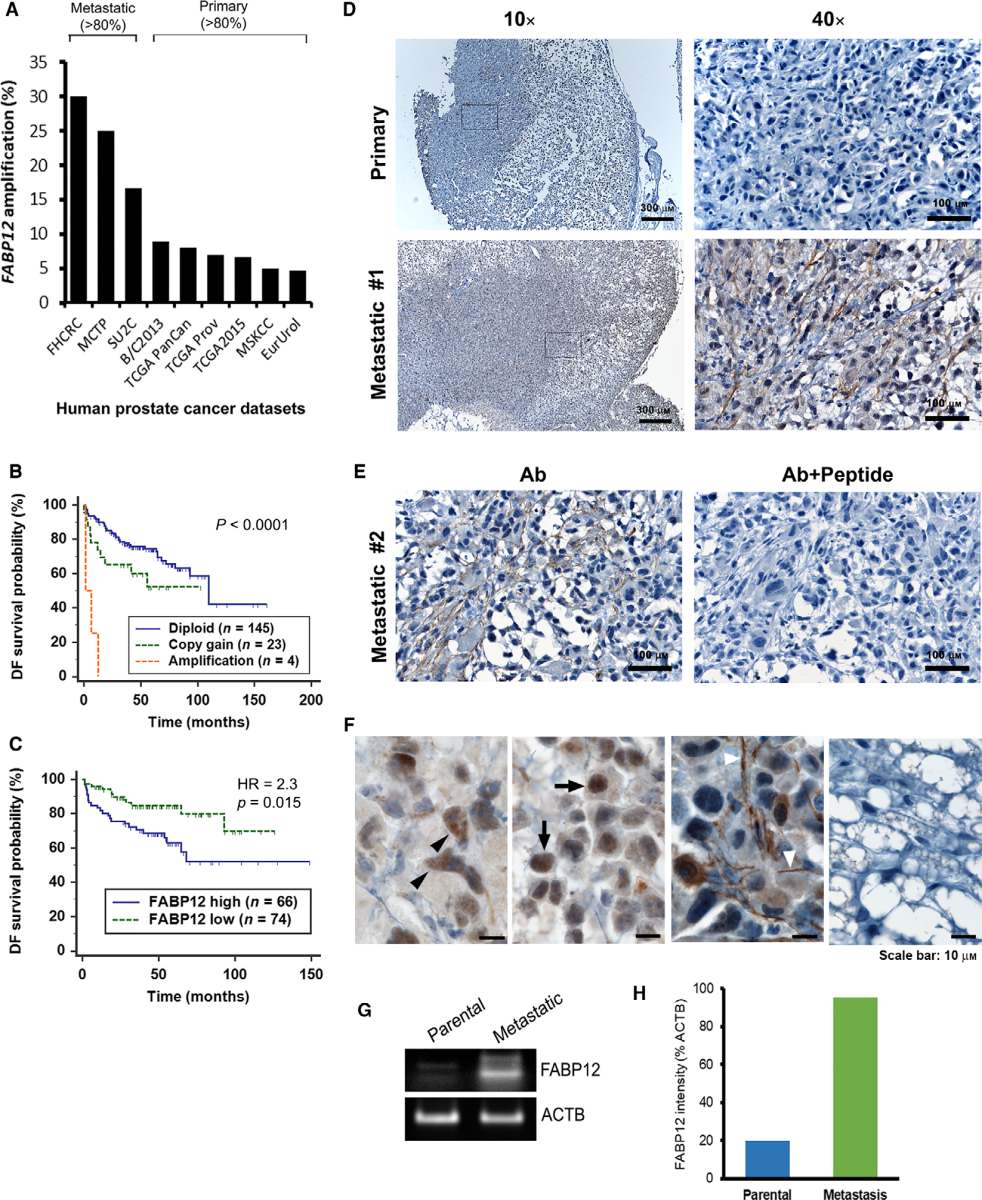
Elevated *FABP12* gene copy numbers and expression levels in metastatic prostate cancer tissues. (A) Increased *FABP12* gene amplification frequency in metastatic PCa patient cohorts compared to primary PCa cohorts. (B–C) Significantly reduced disease‐free (DF) survival probability (log‐rank test) for patients with *FABP12* amplification/copy gain (B) and high mRNA levels (C). The cutoff point for stratifying *FABP12* mRNA levels (*z*‐score = −0.0835) was determined by receiver operating characteristic (ROC) analysis using recurrence status as a classification factor. All datasets were obtained from cBioportal (http://www.cbioportal.org/datasets), and the dataset used for survival analysis was MSKCC, *Cancer Cell* 2010 [48]. *n*, sample size; HR, hazard ratio; *P*, statistical significance level. (D) Primary tumor tissue removed from the flank and axillary lymph nodes of NSG mice injected with PC3 cells and immunostained with anti‐FABP12 antibody shows weak immunoreactivity (upper panels) compared to metastatic tumor tissue (lower panels). (E) The FABP12 immunoreactivity signal observed in a second metastatic tumor is also increased compared to the primary tumor (left panel). Addition of FABP12 peptide as competitor results in complete loss of signal (right panel). (F) Magnified images showing subcellular distribution of FABP12 in xenograft metastatic tumor tissues. (G) Semiquantitative RT‐PCR showing increased *FABP12* RNA levels in a PC3‐induced metastatic xenograft tumors compared to PC3 parental cells. (H) Quantification of *FABP12* signal intensity (relative to actin).

We examined the gene amplification status of *FABP12* in nine human PCa patient cohorts from cBioportal. Three cohorts that consisted mainly of patients with metastatic disease (> 80% of the whole population) showed frequencies of *FABP12* amplification ranging from 17% to 30%. The other six cohorts which consisted mainly of localized tumors (> 80%) had much lower frequencies of *FABP12* amplification (5–8%) (Fig. 1A). We then compared the frequencies of tumors with *FABP12* diploid (CN = 0), copy gain (CN = 1), and amplification (CN ≥ 2) between a primary (MSKCC) and a metastatic (SU2C) cohort. Metastatic PCa had significantly higher frequencies of *FABP12* copy gain (54% vs 15%) and amplification (17% vs 5%) compared to primary PCa, but lower frequencies of *FABP12* diploid tumors (29% vs 80%) (Fig. S1A,B). Kaplan–Meier survival analysis showed that patients with *FABP12* copy gain, amplification, and high mRNA levels have significantly lower recurrence‐free survival probability compared to those with *FABP12* diploid (*P* < 0.0001) and low mRNA levels (*P* = 0.015) (Fig. 1B,C). The dramatic drop in survival probability for patients with *FABP12* amplification may be attributable to the additive effects of other oncogenes residing within the same amplicon [37, 39, 40, 41]. Furthermore, elevated *FABP12* levels were significantly associated with high Gleason scores (Fig. S1C) and metastatic status (Fig. S1D). These results indicate an association between FABP12 and poor prognosis in PCa.

### 3.2 Increased FABP12 levels in metastatic xenograft PCa tumors

We examined *FABP12* RNA levels in four human PCa cell lines (VCaP, PC3, DU145, LNCaP) derived from metastatic tumors using semiquantitative PCR. These cell lines showed weak (VCaP, PC3, and LNCaP) to barely detectable levels of *FABP12* mRNA (DU145) (Fig. S2A). C4.2, an LNCaP subline derived from a mouse xenograft, had slightly higher levels of *FABP12* RNA compared to the parent cell line.

To investigate a possible role for FABP12 in metastasis, we immunostained primary and metastatic mouse PC3 xenograft tumor tissues with anti‐FABP12 antibody. The FABP12 signal was considerably weaker in primary tumors (Fig. 1D, upper panels) compared to metastatic tumors (Fig. 1D—bottom panels; Fig. 1E, left panel). The signal completely disappeared when an FABP12 peptide was used as a competitor for antibody binding (Fig. 1E, right panel). We observed FABP12 in the cytoplasm (Fig. 1F, black arrowhead), nucleus (Fig. 1F, arrow), and cell processes (Fig. 1F, white arrowhead) of the metastatic tumor tissues. The adjacent mouse stroma tissue was FABP12‐negative (Fig. 1F, right most panel). FABP12's subcellular distribution indicates roles in both the cytoplasm and nucleus. We also observed a striking increase in *FABP12* transcript levels in PC3‐derived metastatic tumor tissue compared to PC3 parental cells (Fig. 1G,H). Thus, elevation of FABP12 in our xenograft metastatic tumors mimics the preferential expression of FABP12 in metastatic tumors observed in PCa patient cohorts (Fig. S1D). These results support an association between FABP12 and PCa metastasis.

### 3.3 FABP12 promotes migration and invasion in PCa cells

To investigate the effect of FABP12 expression on cellular properties, we transiently transfected DU145 with HA‐pcDNA3.1 and HA‐pcDNA3.1‐FABP12 expression constructs (Fig. S3A) and examined cell motility using the scratch assay. The relative open area (percentage of the scratched area at time 0) was 67%, 46%, and 36%, respectively, for control cells, and 52%, 27%, and 11%, respectively, for the FABP12‐overexpressing cells after 12, 24, and 36 h (Fig. S3B,C), indicating a role of FABP12 in enhancing cell motility in DU145.

**Fig. 2:**
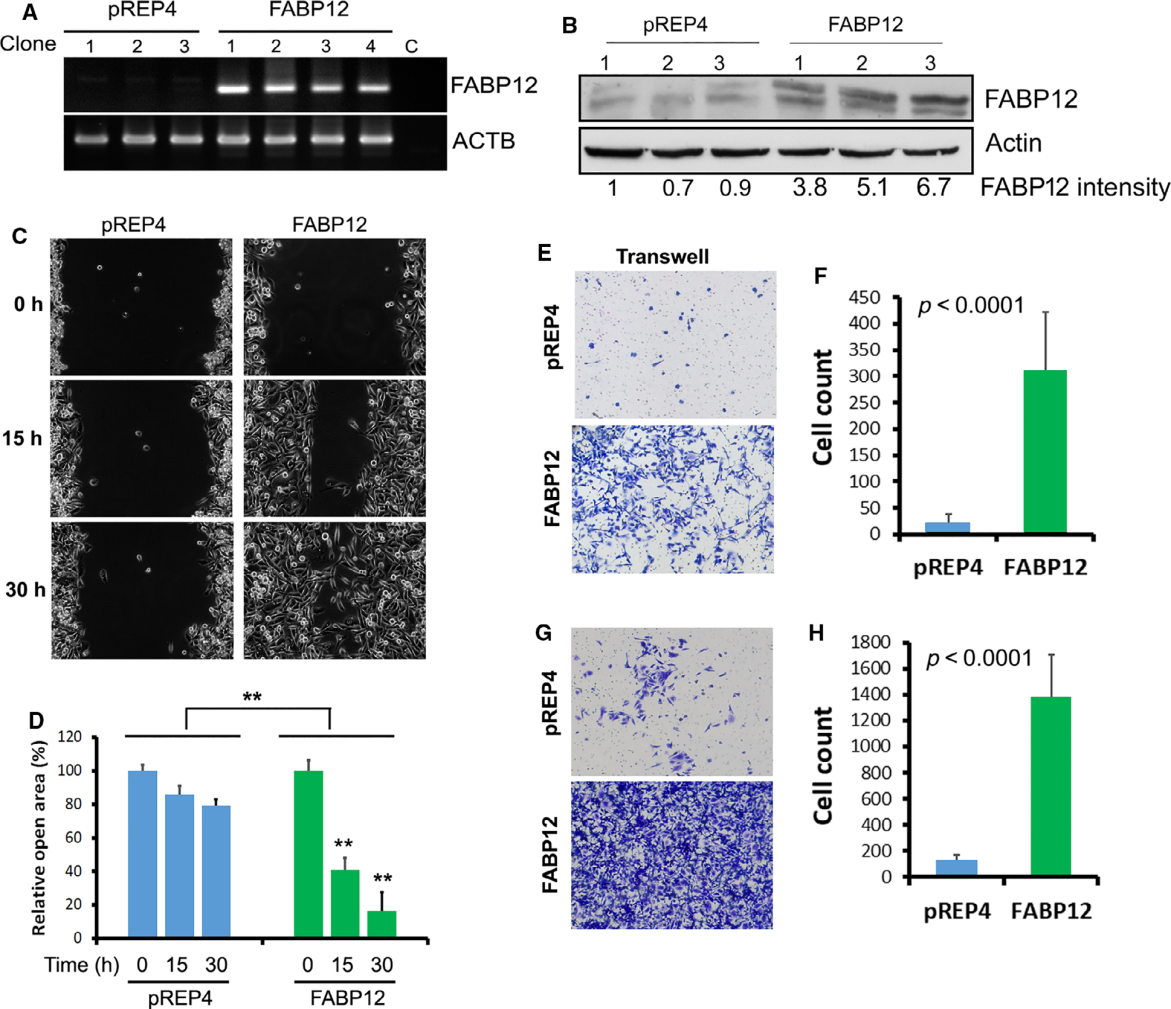
FABP12 promotes cell migration and invasion in prostate cancer cells. (A) Semiquantitative RT‐PCR analysis of *FABP12* RNA levels in PC3 cells with stable ectopic expression of pREP4 empty vector (pREP4) or pREP4‐FABP12 expression construct (FABP12). β‐actin (*ACTB*) was used as the loading control for PCRs. (B) FABP12 protein levels were quantified relative to β‐actin in stably transfected PC3 cell lines. Lane 1 (set at 1) serves as the reference lane. (C) Cell motility of PC3 cells stably transfected with pREP4‐FABP12 constructs (FABP12, right panel) or control (pREP4, left panel), measured using the scratch assay. (D) Open areas relative to the initial *scratched areas* (which were set to 100%) at the designated time points were quantified and analyzed by two‐way ANOVA (*N* = 3). The open area of the scratch was 100%, 86%, and 79% at 0, 15, and 30 h, respectively, for the PC3‐pREP4 cells, compared to 100%, 41%, and 17% for the PC3‐FABP12 cells (*P* < 0.001). (E, F) Representative images (E) and results of Students *t*‐test analysis (F) showing differences in cell migration for PC3 cells stably transfected with pREP4 (control) or pREP4‐FABP12 (FABP12) constructs using the Transwell assay. (G, H) Representative images (G) and results of Student's *t*‐test analysis (H) showing differences in cell invasion for PC3 cells stably transfected with pREP4 (control) or pREP4‐FABP12 (FABP12) constructs using the Matrigel assay. *N* = 3 for each cell line tested for cell migration and invasion. Error bars: SD.

PC3 cells were transiently transfected with HA‐pcDNA3.1 and HA‐pcDNA3.1‐FABP12 expression constructs, and stably transfected with pREP4 (three clonal populations were selected) and pREP4‐FABP12 (four clonal populations selected) expression constructs. Ectopic expression of FABP12 was verified by RT‐PCR (Fig. 2A) and western blotting (Fig. 2B). Both transient and stable FABP12‐expressing PC3 cells showed reduced growth rates, 0.66× (transient) and 0.67× (stable), respectively, compared to control cells (set at 1) 72 h after plating (Fig. S4). Similar to DU145 cells, PC3‐pREP4‐FABP12 showed increased motility compared to control cells. (Fig. 2C,D). Cell migration using the Transwell assay was also significantly increased in PC3‐pREP4‐FABP12 cells compared to PC3‐pREP4 (310 vs 20 cells/field, *P* < 0.0001; 48 h after plating) (Fig. 2E,F). Importantly, there was a ~ 10× increase in the number of invasive PC3‐pREP4‐FABP12 cells compared to control cells using the Matrigel invasion assay (1382 vs 123 cells/field, *P* < 0.0001; 4 days after plating) (Fig. 2G,H).

### 3.4 FABP12 drives epithelial‐to‐mesenchymal transition

**Fig. 3:**
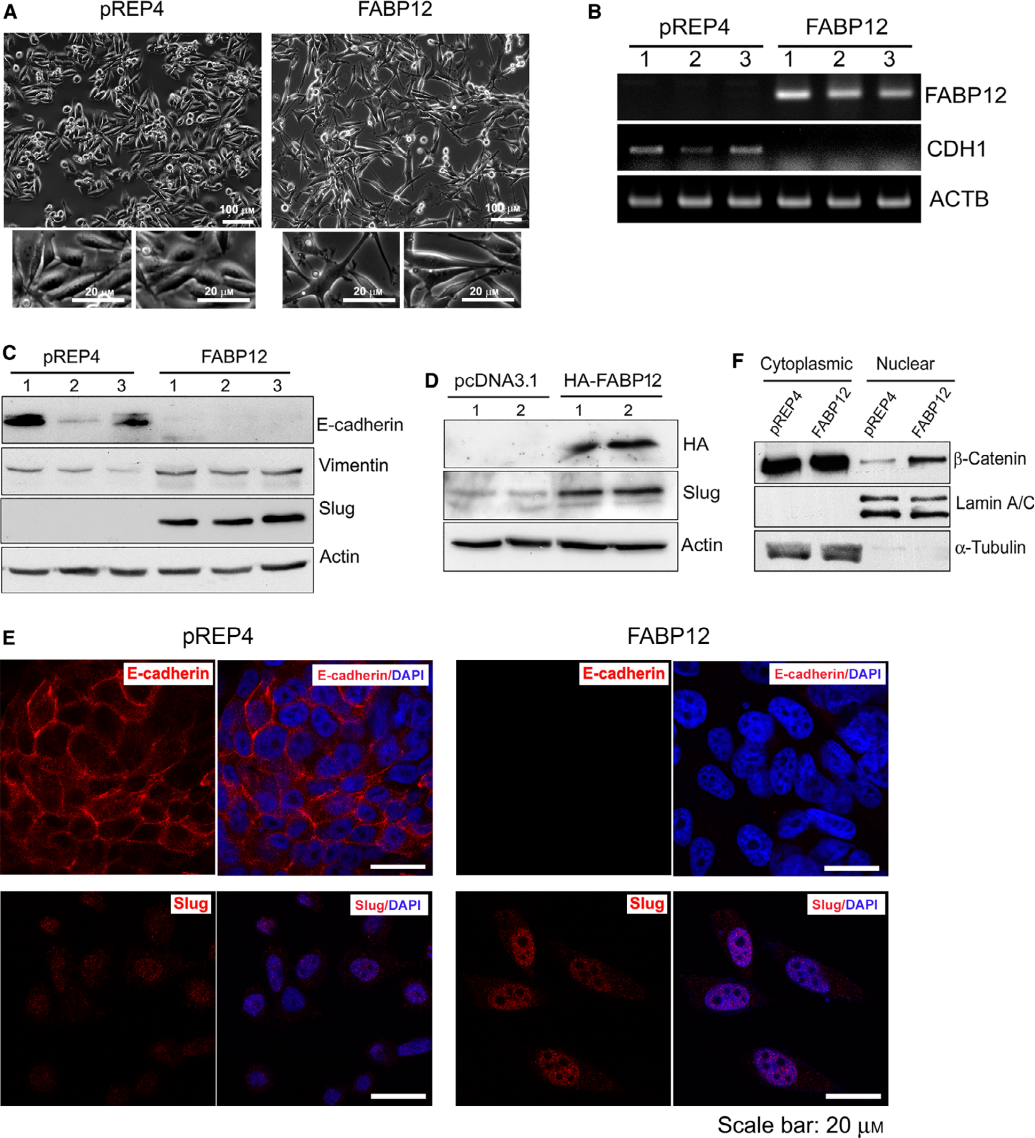
FABP12 induces EMT. (A) Stable ectopic expression of FABP12 induces morphological changes in PC3 cells. FABP12‐expressing cells show an enlarged and fibroblast‐like appearance with elongated processes (right panel, with magnified images from two different regions shown below) compared to the control cells which have a slightly elongated shape (left panel, with magnified images from two different regions shown below) compared to the control cells which have a slightly elongated shape (left panel). (B) Semiquantitative RT‐PCR showing the loss of *CDH1* (encoding E‐cadherin) transcripts in PC3‐pRE4‐FABP12 cells. (C) Western blotting showing the induction of Slug, loss of E‐cadherin, and increased levels of vimentin in PC3‐pREP4‐FABP12 compared to PC3‐pREP4 control cells. (D) Western blotting showing the induction of Slug in PC3 cells transiently transfected with pcDNA3.1‐HA‐FABP12 compared to control cells. (E) Immunofluorescence staining of E‐cadherin and Slug in PC3‐pREP4 control (left panel) and PC3‐pREP4‐FABP12 cells (right panel). (F) Western blotting showing increased levels of β‐catenin in the nucleus of PC3‐pREP4‐FABP12 cells compared to PC3‐pREP4 cells. Human β‐actin, lamin A/C, and α‐tubulin served as loading controls for whole‐cell, nuclear, and cytoplasmic lysates, respectively. *N* = 3.

Epithelial‐to‐mesenchymal transition is associated with invasion and early stages of metastasis [35, 49]. We found that the morphology of PC3 cells dramatically changed upon stable expression of FABP12. The PC3‐pREP4 cells had a roundish shape with short extensions, whereas the PC3‐pREP4‐FABP12 cells were larger and fibroblast‐like with elongated processes (Fig. 3A). This morphology is typical of cells undergoing EMT. Similar morphological changes were also observed in DU145 cells with ectopic expression of FABP12 (Fig. S5A). We further found that ectopic expression of FABP12 resulted in loss of *CDH1* (E‐cadherin) expression, at both the RNA (Fig. 3B) and protein (Fig. 3C) levels. E‐cadherin is a factor critical for maintenance of epithelial cell structure [50]. Notably, there was a much higher negative correlation between *FABP12* and *CDH1* mRNA levels in metastatic tumors (*r* = −0.66, *P* = 0.002) than in primary tumors (*r* = −0.25, *P* = 0.004). FABP12 overexpression also resulted in upregulation of vimentin, a mesenchymal marker [51], and induction of Slug, a key transcription factor that triggers EMT and directly suppresses E‐cadherin [52, 53] (Fig. 3C). Induction of Slug in FABP12‐expressing cells was also observed in two independent transient transfection experiments (Fig. 3D). FABP12‐induced loss of E‐cadherin and upregulation of Slug were verified by immunofluorescence staining of stable PC3 control and FABP12‐overexpressing cell lines (Fig. 3E). Finally, we immunostained our xenograft PCa tumor tissue sections with EMT markers. In contrast to primary tumors, and in agreement with our cell line data, we observed loss of E‐cadherin, and elevation of Slug and fibronectin in metastatic tumors (Fig. S6).

Next, we analyzed the subcellular localization of β‐catenin, an E‐cadherin‐interacting partner critical for maintenance of the epithelial phenotype. Loss of E‐cadherin facilitates β‐catenin release and translocation into the nucleus, which in turn promotes EMT through nuclear signaling [54]. We observed an increase in β‐catenin nuclear accumulation, concomitant with E‐cadherin loss in PC3‐pREP4‐FABP12 cells compared to control cells. There were no obvious changes in the cytoplasmic levels of β‐catenin (Fig. 3F). These combined findings suggest a role for FABP12 as a driver of EMT in PCa cells.

**Fig. 4:**
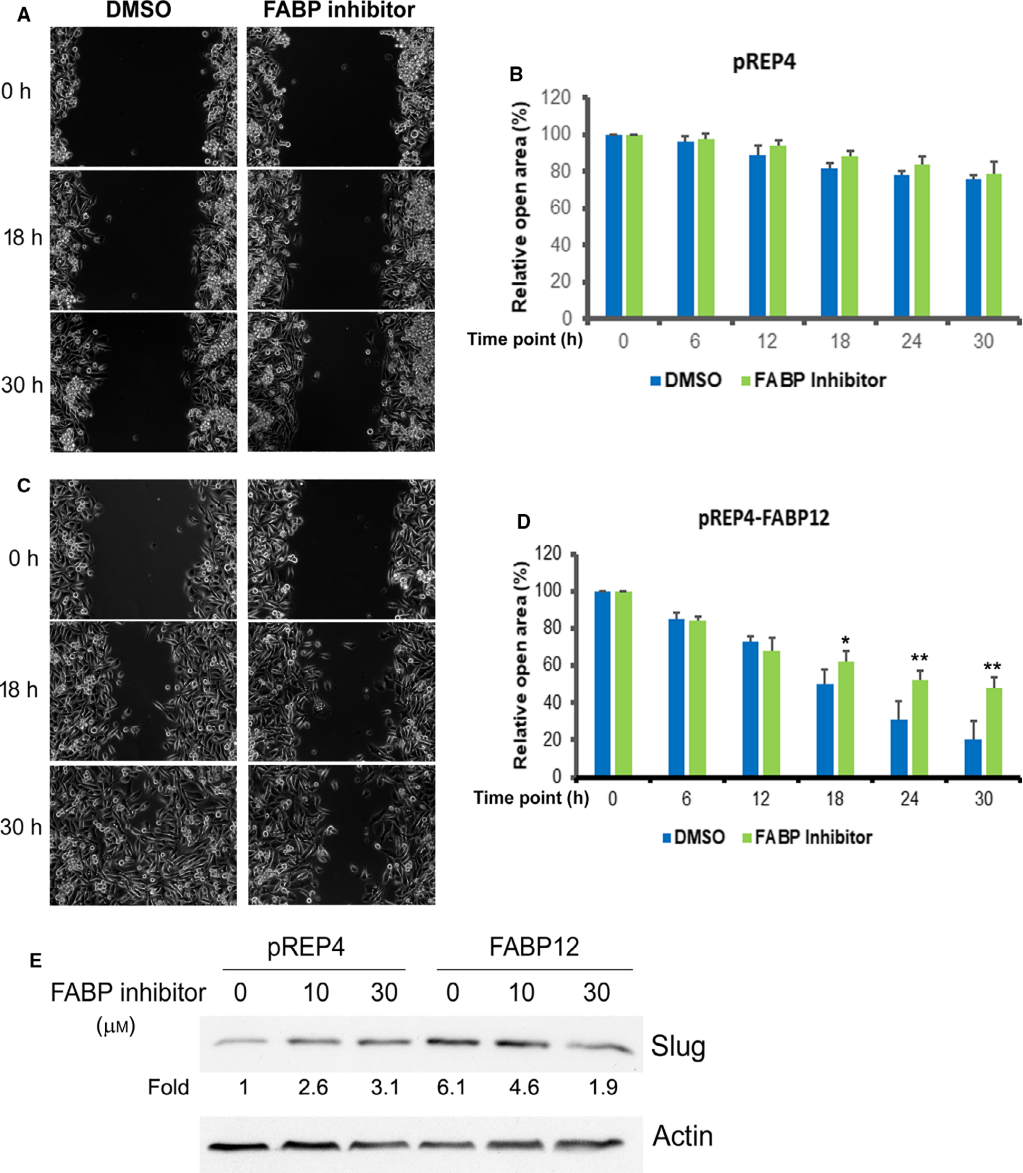
Effect of FABP inhibitor (T4B) on FABP12‐induced cell motility and Slug expression. PC3 cells stably transfected with control vector (pREP4) (A, B) or FABP12 expression construct (pREP4‐FABP12) (C, D) were treated with DMSO or 30 μm T4B and subjected to the scratch assay. Cell motility was recorded as change in the scratch open area over a 30‐h period. Left panels (A) and (C) are phase‐contrast microscopy images showing changes in the scratched area at the indicated time points for PC3 control and PC3‐FABP12 cells, respectively. The quantified changes in the open area (percent relative to the initial scratch) are shown in the panels on the right (B, D). Statistical analyses were done using Student's *t*‐test. Error bar: SD. *P < 0.05; **P < 0.01. (E) FABP12‐induced Slug expression is reduced upon FABP inhibitor treatment in a dose‐dependent manner.

To examine cell motility, we carried out the scratch assay using stable PC3‐pREP4 control and PC3‐pREP4‐FABP12 cells treated with the FABP inhibitor, CAS300657‐03‐8 (T4B). While T4B has previously been used to block FABP4 activity [55], our protein–ligand binding modeling analysis using COACH [56] identified T4B as a top‐ranked ligand for FABP12 with a TM‐score (indicative of structure similarity) of 0.94. We observed significant FABP inhibitor‐dependent reduction in cell motility in FABP12‐expressing PC3 cells but not in control cells which express abundant FABP4 and FABP5 (Fig. 4A–D and Fig. S2B), suggesting that FABP12, rather than FABP4 and FABP5, is the predominant driver for cell motility for these cell models. Semiquantitative PCR analysis showed that ectopic expression of FABP12 did not affect the levels FABP4, FABP5, and FABP9, paralogs known to be expressed in PCa cells (Fig. S2B). These results suggest that the effect of T4B on PCa cell motility is due to specific inhibition of FABP12.

Furthermore, FABP12‐induced Slug expression was reduced in a dose‐dependent manner upon FABP inhibition (T4B) in PC3‐pREP4‐FABP12 cells, but not control cells (Fig. 4E), suggesting that T4B‐mediated inhibition of cell motility may be through inhibition of Slug activity. Depletion of Slug in FABP12‐expressing PC3 cells using siRNAs targeting *SNAI2* (Slug) caused reversal of the increase in cell motility associated with FABP12 expression (Fig. S7). These results suggest that the EMT‐inducing factor Slug is a main downstream effector of FABP12 in triggering cell motility and EMT.

### 3.5 FABP12 induces PPARγ activation

PPARγ, a nuclear receptor implicated in fatty acid signaling and lipid metabolism, has been reported to be a driver of metastasis in PCa [57]. We therefore analyzed the effect of FABP12 on PPARγ activation (measured by binding to the peroxisome proliferator response element, PPRE) using the gel shift assay. We first transiently transfected PC3 cells with pcDNA3.1‐HA, pcDNA3.1‐HA‐FABP12, or pcDNA3.1‐HA‐FABP7. FABP7 is not normally expressed in PC3 cells but has been implicated in the regulation of PPARγ activity [58]. Compared to control cells, we observed an increase in the intensity of the PPRE DNA–nuclear protein complex (arrow) in PC3 cells transfected with FABP12 but not with FABP7 expression constructs (Fig. S8, left panel). The supershift assay revealed a slower‐migrating band (arrowhead) with anti‐PPARγ, but not anti‐PPARα or anti‐PPARβ/δ antibodies, in FABP12‐expressing PC3 cells (Fig. S8, right panel). These results indicate that PPARγ is part of the PPRE–nuclear protein complexes observed in the gel shifts.

**Fig. 5:**
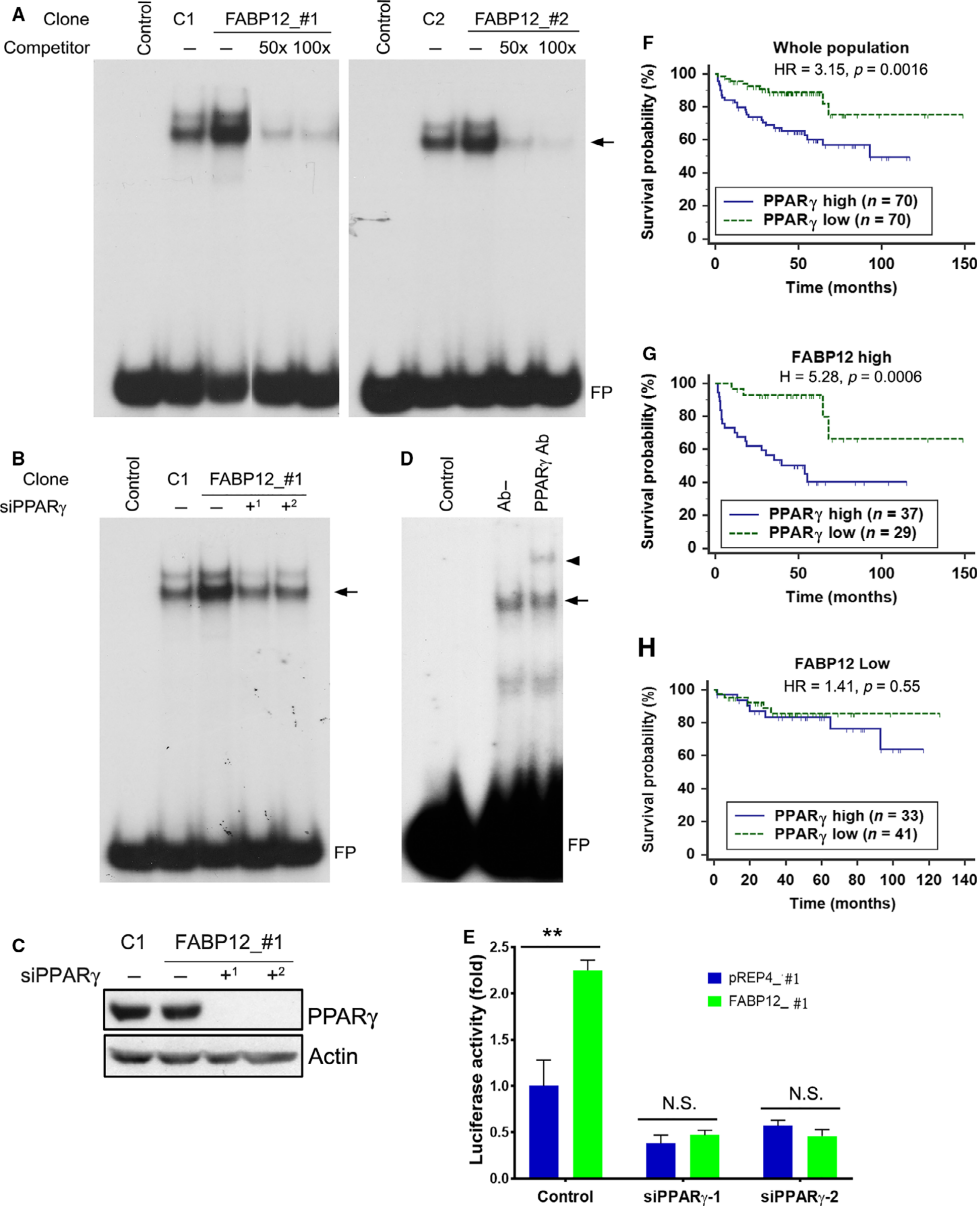
FABP12 facilitates activation of PPARγ and determines PPARγ prognostic significance. (A) Nuclear extracts prepared from two stable PC3‐pREP4‐FABP12 clonal populations (FABP12_#1, FABP12_#2; lanes 3) enhanced the formation of nuclear protein–PPRE complexes (arrow) compared to control PC3‐pREP4 clonal populations (C1, C2; lanes 2) in an electrophoretic mobility shift assay (EMSA). Excess unlabeled PPRE probe (50× excess in lanes 4 and 100× excess in lanes 5) effectively competed with the radiolabeled probe, resulting in considerably reduced protein–PPRE complexes. FP denotes free probe. (B) PPARγ depletion in stable PC3‐pREP4‐FABP12 transfectants using two different siRNAs reduced protein–PPRE complex formation (arrow) (compare lane 3 with lanes 4 and 5). (C) Western blot showing reduced levels of PPARγ in cells transfected with two PPARγ siRNAs, with actin serving as the loading control. (D) A supershifted protein–PPRE complex (arrowhead) is observed upon addition of anti‐PPARγ antibody (lane 3). No antibody was added to lane 2 (Ab−). (E) PPRE‐driven luciferase activity in PC3 control (pREP4) and PC3‐FABP12 overexpression (FABP12) stable cell lines transfected with scrambled (control) or PPARγ‐specific siRNAs. Statistical analysis was done using Student's *t*‐test. (F–H) High levels of *PPARγ* mRNA are significantly correlated with a worse prognosis in a PCa patient cohort (MSKCC dataset described in [48]) (F). This prognostic significance is markedly increased in the subpopulation of PCa patients with high levels of *FABP12* mRNA (G), but eliminated in the subpopulation with low *FABP12* levels (H). Log‐rank test was used for patient survival analysis. The cutoff point for stratifying *PPARγ* mRNA levels was determined by receiver operating characteristic (ROC) analysis using disease‐free status as a classification factor. HR, hazard ratio; *n*, sample size. Error bar: SD.

We then repeated the gel shift assay using nuclear extracts from PC3‐pREP4 and –pREP4‐FABP12 stable cell lines. We observed PPRE DNA–protein complexes (arrow) in all four PC3 clonal populations tested. However, the signal intensity was markedly increased in PC3‐pREP4‐FABP12 (FABP12_#1 and #2) cells compared to control cells (C1 and C2). Addition of 50× and 100× excess unlabeled probe to the binding reaction effectively competed with the labeled probe, with little to no signal detected, indicating probe‐specific interactions (Fig. 5A).

To further address FABP12‐mediated PPARγ‐specific binding to PPRE, we carried out gel shift experiments with nuclear lysates from PC3‐pREP4 and PC3‐pREP4‐FABP12 cells with or without PPARγ depletion (Fig. 5B,C). Again, increased intensity of the binding complex was observed in PC3‐pREP4‐FABP12 cells (lane 3) compared to PC3‐pREP4 cells (lane 2). PPARγ depletion using two different siRNAs resulted in reduced protein‐DNA binding, with signal intensities similar to those observed in control PC3 cells (lanes 4 and 5). Supershift experiments with anti‐PPARγ antibody resulted in a slower‐migrating band (arrowhead), in support of the presence of PPARγ in the binding complex (Fig. 5D). Next, we used a PPRE‐driven luciferase reporter construct to further investigate the effect of FABP12 on PPARγ activation. Ectopic expression of FABP12 in PC3 cells increased PPRE‐driven luciferase expression/activity by 2.25‐fold (pREP4 vs FABP12, *P* < 0.01) (Fig. 5E). The effect of FABP12 on luciferase activity was substantially reduced (~ 5‐fold) upon depletion of PPARγ (Fig. 5E). Similar to PC3 cells, ectopic expression of FABP12 in DU145 cells increased PPRE‐driven luciferase activity (Fig. S5B), despite reduction in cell growth (Fig. S5C). Together, these results indicate a role for FABP12 in the activation of PPARγ.

### 3.6 PPARγ promotes progression‐related properties in FABP12‐expressing PCa cells

We analyzed the prognostic significance of PPARγ using a PCa dataset from cBioportal [48]. We found that patients with high levels of *PPARγ* had a significantly worse prognosis compared to those with low *PPARγ* levels in the whole population (HR = 3.15, *P* = 0.0016) (Fig. 5F). Notably, this effect was further magnified in the subpopulation of patients with high levels of *FABP12* (HR = 5.28, *P* < 0.0006) (Fig. 5G), but became insignificant in the population with low levels of *FABP12* (HR = 1.41, *P* = 0.55) (Fig. 5H).

**Fig. 6:**
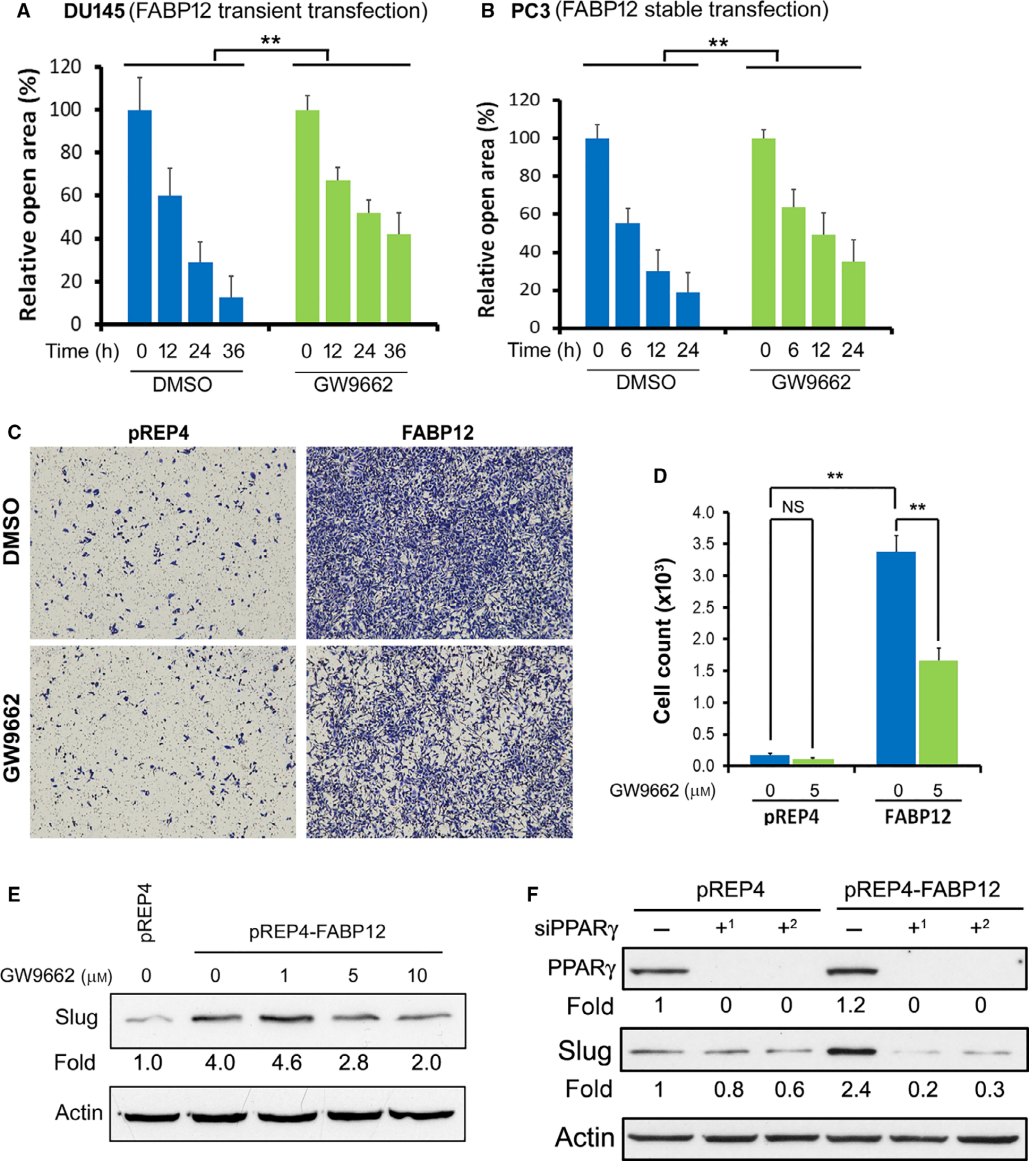
Blocking PPARγ reverses the effects of FABP12 on cell migration and Slug induction. (A, B) The scratch assay shows that treatment with the PPARγ inhibitor GW9662 reduces cell motility (increased relative open area) in DU145 cells transiently transfected with pcDNA3.1‐HA‐FABP12 compared to control transfectants (A) and PC3 cells stably transfected with pREP4‐FABP12 compared to control transfectants (B). Two‐way ANOVA was used for statistical analysis. (C, D) The Transwell assay shows that treatment of stable PC3‐pREP4‐FABP12 transfectants with PPARγ inhibitor (GW9662) reduces cell migration. Images are shown in (C) and cell counts in (D). Statistical analysis was carried out using Student's *t*‐test. (E, F) Western blot showing reversal of Slug induction in PC3‐pREP4‐FABP12 cells upon PPARγ inhibition with GW9662 (E) or PPARγ depletion using two different siRNAs (F). **P < 0.01. *N* = 3. Error bar: SD.

We next asked whether PPARγ underlies the FABP12‐mediated increases in motility, migration, and invasiveness observed in our previous experiments. Our scratch assay showed that transiently transfected DU145‐HA‐pcDNA3.1‐FABP12 cells treated with vehicle control had relative open areas of 59%, 29%, and 13%, at 12, 24, and 36 h, respectively, with significant increases to 67%, 52%, and 42% when cells were treated with GW9662, a potent PPARγ inhibitor (Fig. 6A). A similar trend (56%, 31% and 20% vs 65%, 51% and 36%) was observed in PC3 cells stably transfected with pREP4‐FABP12 (Fig. 6B).

Stable PC3‐pREP4‐FABP12 cells treated with vehicle control showed a 21‐fold increase in cell migration compared to control cells using the Transwell assay. Importantly, when cells were treated with PPARγ inhibitor GW9662, we observed a significant decrease (~ 2‐fold) in cell migration in PC3‐pREP4‐FABP12 cells, but not in PC3‐pREP4 cells (Fig. 6C,D). Similar results were observed upon depletion of PPARγ with siRNA (Fig. S9). Finally, inhibition of PPARγ with GW9662 (Fig. 6E) or depletion of PPARγ with siRNAs (Fig. 6F) in stable PC3‐pREP4‐FABP12 cells reversed FABP12‐dependent upregulation of Slug. However, PPARγ depletion in PC3‐pREP4 control cells resulted in only a slight reduction in Slug levels (Fig. 6F). These results support an important role for PPARγ in mediating the function of FABP12 in PCa progression.

### 3.7 The interplay of FABP12 and PPARγ modulates intracellular lipid accumulation and energy metabolism

**Fig. 7:**
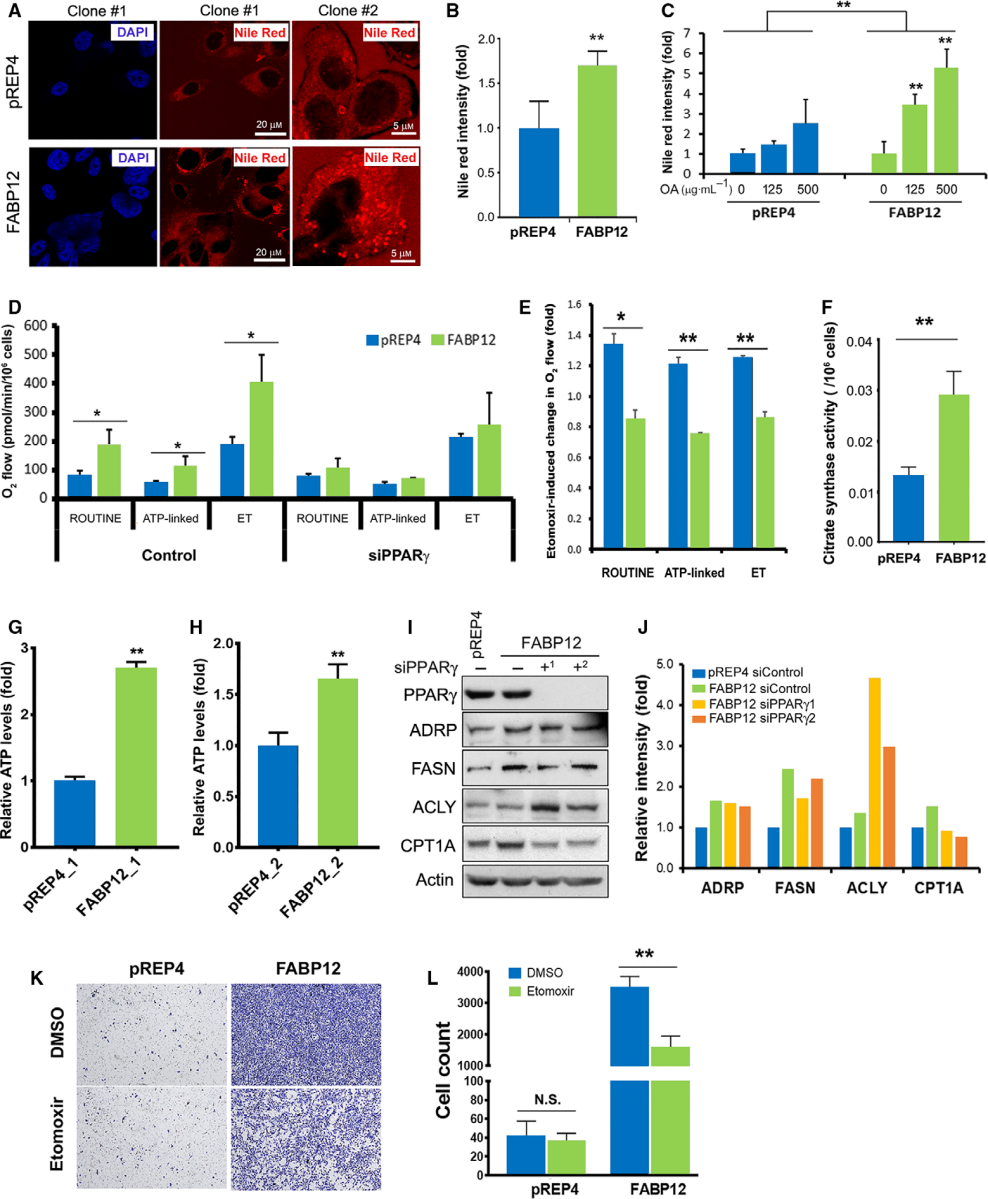
FABP12 expression enhances cellular lipid accumulation and mitochondrial fatty acid β‐oxidation. (A) Confocal microscopy images of Nile Red staining of lipid droplets in PC3 control cells—clone #1 (pREP4, upper panel) and PC3 cells stably expressing FABP12—clone #1 (lower panel). The panels on the right show magnified images of PC3‐pREP4—clone #2 and PC3‐pREP4‐FABP12 clone #2. DAPI was used as a nuclear stain for PC3—clones #1 (left panels). (B) Quantification of Nile Red staining of lipid droplets in stable PC3 control and FABP12‐expressing cells. (C) Lipid droplet accumulation in stable PC3 control and FABP12‐expressing cells cultured in medium supplemented with oleic acid. Two‐way ANOVA was used for statistical analysis. (D) Oxygen consumption by mitochondria of stable PC3 control and FABP12‐expressing cells. Significantly increased ROUTINE, ATP‐linked, and ET (maximum) O_2_ consumption was observed in FABP12‐expressing cells. This increase in O_2_ consumption was no longer observed upon PPARγ depletion. (E) O_2_ consumption in ROUTINE, ATP‐linked, and ET states was all decreased in PC3 cells stably transfected with pREP4‐FABP12 (FABP12) compared to control cells (pREP4) upon treatment with 100 μm etomoxir, a CPT1 inhibitor. (F) Ectopic expression of FABP12 induces significantly higher activity of citrate synthase, the rate‐limiting enzyme for the Kreb's cycle. (G, H) Increased ATP levels were observed in two clonal populations of stably transfected PC3‐pREP4‐FABP12 cells compared to control PC3‐pREP4 cells. (I, J) Effect of FABP12 ectopic expression and PPARγ depletion on expression of key enzymes related to lipid metabolism. (K, L) Representative images (K) and results (L) showing the effects of FABP12 expression and etomoxir treatment on cell migration using the Transwell assay. Statistical analyses in B, D, E, F, G, H, J, and L were carried out using Student's *t*‐test. *P < 0.05; **P < 0.01. Error bar: SD.

To address lipid accumulation, we stained stably transfected PC3‐pREP4 and PC3‐pREP4‐FABP12 cells with Nile Red, a lipophilic dye that measures intracellular lipid droplets. Lipid droplets are site of lipid storage and have been associated with desaturation of fatty acids and cancer growth and survival [59, 60]. We observed a 1.7× increase of Nile Red staining intensity in PC3‐pREP4‐FABP12 cells compared to control cells (*P* < 0.01) (Fig. 7A,B). We also observed marked increase in ADRP (a lipid droplet marker) immunoreactivity in PC3‐pREP4‐FABP12 cells compared to control cells (Fig. S10).

Next, we asked whether FABP12 affects uptake of exogenous fatty acids. We found that supplementation of oleic acid (a monounsaturated omega‐9 fatty acid) did not significantly change lipid droplet accumulation in PC3 control cells, but significantly increased lipid droplets in PC3‐pREP4‐FABP12 cells in a dose‐dependent manner (Fig. 7C). These results suggest an active role for FABP12 in the uptake of fatty acids leading to increased lipid droplet accumulation.

We then examined a possible role for FABP12 in energy production using the Oxygraph‐2k system. Cellular energy, in the form of ATP, can be generated via either anaerobic glycolysis in the cytoplasm or oxidative phosphorylation in the mitochondria. The rate of oxygen consumption by cells is a measure of mitochondrial oxidative phosphorylation. In addition to pyruvate (derived from glucose), fatty acids can also serve as electron donors to produce ATP through oxidative phosphorylation. Here, we measure the respiration of ROUTINE (total cellular oxygen consumption in the physiological coupling state), ATP‐linked (ROUTINE strictly coupled to phosphorylation of ADP after subtracting the nonphosphorylating LEAK respiration), and ET (maximum oxygen flux as the determinant of electron transfer capacity) states. Compared to control cells, FABP12‐expressing PC3 cells showed significant increases in ROUTINE (84 vs 191, *P* = 0.02), ATP‐linked (Routine‐LEAK) (56 vs 116, *P* = 0.03), and ET (maximum) state (188 vs 408, *P* = 0.02) respiration (oxygen flow in pmol·min^−1^·10^−6^ cells). Of note, this FABP12‐dependent effect was no longer observed upon PPARγ depletion (79 vs 109, *P* = 0.24 for ROUTINE; 52 vs 70, *P* = 0.12 for ATP‐linked; 213 vs 257, *P* = 0.73 for ET states). These results demonstrate the importance of FABP12 for increased energy production, which is in turn dependent on PPARγ (Fig. 7D).

To test whether FABP12 facilitates mitochondrial energy production from fatty acids, we analyzed mitochondrial respiration in stable PC3‐pREP4 and PC3‐pREP4‐FABP12 cells cultured in the presence or absence of a potent inhibitor of CPT1 (etomoxir), a rate‐limiting enzyme mediating fatty acid β‐oxidation in mitochondria [61]. CPT1 inhibition in PC3‐pREP4‐FABP12 cells resulted in a decrease in ROUTINE (0.85×), ATP‐linked (0.76×), and ET state (0.86×) O_2_ consumption compared to cells cultured in the absence of etomoxir (Fig. 7E). In contrast, CPT1 inhibition in PC3‐pREP4 cells enhanced ROUTINE (1.34×), ATP‐linked (1.21×), and ET state (1.26×) O_2_ consumption (Fig. 7E). The difference in fatty acid oxidation observed in PC3‐pREP4‐FABP12 compared to control cells is significant (*P* = 0.015) for ROUTINE, and very significant for both ATP‐linked (*P* = 0.004) and ET state (*P* = 0.004) mitochondrial O_2_ consumption (Fig. 7E). Together these results indicate a role for FABP12 in promoting lipid accumulation and lipid utilization for energy production in PCa cells. We then analyzed the activity of citrate synthase (CS), the first and rate‐limiting enzyme in the Kreb's cycle. Ectopic expression of FABP12 in PC3 cells resulted in a 2.2‐fold induction in CS activity (*P* < 0.01) compared to control cells (Fig. 7F), supporting a critical role for FABP12 in mitochondrial energy production.

We also measured ATP levels in our cell models. ATP levels in two independent pREP4‐FABP12 clones were increased by 2.7‐fold (Fig. 7G, *P* < 0.0001) and 1.7‐fold (Fig. 7H, *P* = 0.004), respectively, compared to their corresponding control cells (pREP4_1 and pREP4_2). Unexpectedly, we did not observe significant changes in ATP levels upon PPARγ depletion in either pREP4 control or pREP4‐FABP12 cells (data not shown). We postulate that this is the result of an overall balanced energy supply and demand in FABP12‐expressing PCa cells, with the FABP12‐PPARγ pathway stimulating mitochondrial oxidation for ATP production, while at the same time triggering processes requiring ATP production such as EMT, cell motility, and invasion.

Next, we examined the effect of PPARγ depletion on several key enzymes in cellular fatty acid accumulation and utilization. We observed upregulation of ADRP (a lipid droplet marker), FASN (fatty acid synthase), and CPT1A (rate‐limiting enzyme for conversion of fatty acids from acyl‐coA chains to acyl‐carnitine for utilization as energy source in mitochondria; inhibited by etomoxir) in cells with ectopic expression of FABP12 (Fig. 7I,J). PPARγ depletion reduced CPT1A expression, but had no effect on ADPR and FASN in FABP12 expressing cells. Interestingly, the expression of ACLY, an ATP citrate lyase responsible for the conversion of citrate to acetyl‐CoA, a key step in fatty acid biosynthesis, was not affected by FABP12 expression, but upregulated upon PPARγ knockdown (Fig. 7I,J). These results suggest that the FABP12‐PPARγ pathway primarily drives fatty acid β‐oxidation in mitochondria, with FABP12 playing a PPARγ‐independent role in the induction of *de novo* fatty acid synthesis and lipid storage.

Finally, we examined whether the increase in PCa cell migration observed upon FABP12 expression was dependent on fatty acid utilization in mitochondria. When the transfer of fatty acids into mitochondria was blocked by etomoxir, a potent CPT1 inhibitor, there was a significant reduction (*P* < 0.0001) in the migration of PC3‐FABP12‐expressing cells (Fig. 7K,L). There was no effect on PC3 control cell migration. These results directly implicate FABP12 in fatty acid beta‐oxidation‐driven PCa cell migration.

## 4 Discussion

Prostate cancer is invariably fatal once it metastasizes [62]. Lipid accumulation and metabolic reprogramming have previously been documented in metastatic PCa [15, 63, 64]. In this study, we identify an intracellular lipid transporter gene, *FABP12*, which is preferentially amplified and overexpressed in metastatic PCa and correlates with poor clinical outcomes. FABP12 expression promotes properties associated with cancer progression such as EMT conversion, invasion, and metastasis. In addition, FABP12 expression in PCa cells increases fatty acid uptake, lipid droplet accumulation, and energy production from fatty acids. This is the first report demonstrating a role for a FABP in regulating energy production in PCa cells, and an association between FABP12 expression, EMT and increased lipid bioenergetics. Furthermore, our results indicate that EMT and lipid bioenergetic alterations can both be attributed to FABP12‐mediated activation of PPARγ. Based on our findings, we propose that increased FABP12 expression represents one of the major adaptations of PCa cells required to trigger a pro‐invasive molecular pathway and to satisfy the increased energy demands associated with tumor expansion and metastasis (Graphical abstract).

FABP12‐mediated increases in cell motility, cell migration, and invasion are accompanied by EMT, a process that turns epithelial cells into migratory and invasive mesenchymal cells. As such, EMT is regarded as a critical priming event for metastasis [35]. There is increasing evidence suggesting a link between EMT and metabolic reprogramming (particularly lipid metabolic reprogramming) in cancer [8, 35, 63]. Thus, fatty acids may not only serve as energy sources, but also be involved in protein modification, cell membrane remodeling, and cell signaling, processes that are essential for EMT [8, 63]. In support of these expanded roles for fatty acids, others have demonstrated that PCa cells undergoing EMT show significant increases in lipid accumulation [65]. FABP12‐induced expression of EMT‐promoting Slug, elevation in mitochondria respiration, and increases in cell motility and migration were either reversed, or partially reversed, upon depletion and/or inhibition of PPARγ, pointing to a role for PPARγ in mediating FABP12 functions. PPARs primarily regulate the expression of genes involved in lipogenesis and energy homeostasis [66].

PPARγ is generally believed to be a tumor suppressor as numerous *in vitro* studies show that PPARγ agonists (for instance thiazolidinediones) are able to inhibit tumor cell growth and/or induce differentiation in many different cancers [21, 67]. However, more recent mechanistic studies suggest that many of PPARγ's previously reported anti‐cancer properties may actually be PPARγ‐independent due to off‐target effects of its synthetic agonists [67, 68]. In PCa, PPARγ is overexpressed and its levels are positively correlated with tumor grade and stage [57]. In particular, PPARγ has recently been shown to drive PCa progression/metastasis [57]. In light of our study, we suggest that targeting FABP12, which resides upstream of PPARγ, may delay or prevent PCa progression.

A number of FABPs are expressed in PCa cells, including FABP4, FABP5, and FABP9 [29]. The biological actions of these FABPs in promoting PCa progression appear to be synergic rather than redundant and mediated through distinct pathways. The most extensively studied FABP in PCa, FABP5, promotes PCa cell and/or xenograft tumor growth, with inhibition of both PPARβ/δ and PPARγ suppressing these effects [30, 33, 69, 70, 71, 72, 73]. FABP5 has been proposed to promote PCa metastasis by triggering angiogenesis (VEGF induction) [71, 74] and fatty acid synthesis [33]. FABP4 is secreted from both PCa cells and adipocytes surrounding PCa tumors, and is believed to either shuttle fatty acids between adipocytes and tumor cells [75] or act as an inducer of MMPs from cancer cells and cytokines (e.g., IL‐6, IL‐8) from stroma cells to promote invasion and metastasis [34]. Elevated levels of FABP4 have been reported in PCa bone metastasis in an obese mouse model [76]. Surprisingly, FABP4 expression is downregulated in primary PCa biopsies or cell lines compared to normal tissues/cell lines [77, 78], and ectopic expression of FABP4 in PCa cells causes apoptosis [79]. Increased levels of FABP9 in PCa have been shown to have prognostic significance, with FABP9 depletion resulting in reduced cell invasion [80]. Unlike FABP12 shown here, none of these FABPs have been shown to act as an activator of the EMT pathway or a mediator of lipid‐related energy production in PCa cells.

FABP12 is expressed at very low levels in PCa cell lines. Its ectopic expression in PCa cells has dramatic effects on cellular properties including increased migration and invasion, but significantly reduces rates of proliferation. As cells in culture are naturally selected for rapid proliferation rates, we believe that tissue culture conditions may select for low levels of FABP12. This idea is in keeping with our observation that FABP12 levels are induced in metastatic tumors derived from a mouse xenograft PC3 model and support the ‘Go‐or‐Grow’ hypothesis proposed for tumor invasion [81]. Thus, FABP12 may be at the apex of a critical oncogenic axis leading to PCa metastasis: FABP12 amplification/overexpression → PPARγ activation → dysregulation of lipid metabolism → EMT and increase motility, migration and invasion.

PPARγ and androgen receptor are both ligand‐activated nuclear receptors with important roles in normal prostate development and cancer progression. A number of studies suggest a bidirectional interaction between AR and PPAR, with each receptor influencing the expression and/or activity of the other within prostatic tissues or tumor cells [68, 82]. For example, ligand‐induced activation of AR is significantly reduced upon treatment with PPARγ agonist pioglitazone and troglitazone in LNCaP cells [83], whereas PPARγ expression and/or activation is inhibited or enhanced by increasing or suppressing AR signaling in both castration‐resistant and sensitive PCa cell models [84]. We speculate that overexpression of FABP12 and the consequent activation of the PPARγ pathway may contribute to castration resistance and metastasis in advanced PCa. Future work will focus on the crosstalk between the FABP12‐FA‐PPARγ pathway and androgen signaling.

## 5 Conclusions

We have identified an intracellular lipid‐binding protein (FABP12) that is preferentially amplified and expressed in metastatic PCa tumors. We provide evidence that FABP12 drives aggressive properties in PCa cells by promoting EMT, lipid accumulation, and utilization of fatty acids for mitochondrial energy production. Our data indicate that FABP12 functions upstream of PPARγ, a lipogenic transcription factor implicated in PCa metastasis [57]. Future work will involve targeting FABP12 to investigate its effect on PCa metastasis.

## Conflict of interest

The authors declare no conflict of interest.

## Author contributions

R‐ZL and RG conceived and designed the study. R‐ZL carried out experiments, analyzed the data, and drafted the manuscript. SJ, W‐SC, X‐HY, XX, and WHH carried out experiments. DD, DDG, and RBM designed and carried out the mouse xenograft tumor experiments. HL was involved in the design and data interpretation of the mitochondria respiration analysis. RG and HL edited the manuscript. RG is the recipient of the research grant from Prostate Cancer Canada. All authors reviewed and approved the manuscript.

### Peer Review

The peer review history for this article is available at https://publons.com/publon/10.1002/1878‐0261.12818.

## Supporting information


**Fig. S1.** Association of increased FABP12 gene copy numbers and mRNA levels with PCa metastasis and Gleason scores.
**Fig. S2.** RT‐PCR analysis of FABP transcripts in prostate cancer cell lines.
**Fig. S3.** FABP12 promotes cell migration in DU145.
**Fig. S4.** Effect of transient and stable expression of FABP12 on PC3 cell proliferation.
**Fig. S5.** Effects of FABP12 overexpression on cell morphology, growth and PPAR activation.
**Fig. S6.** Immunohistochemical staining of EMT markers in PC3‐derived xenograft primary and metastatic tumor tissues.
**Fig. S7.** FABP12‐induced cell motility in PC3 cells is reduced upon SNAI2 (Slug) depletion.
**Fig. S8.** Effect of transient expression of FABP12 on PPARγ transactivation in PC3 cells.
**Fig. S9.** PPARγ depletion attenuates FABP12‐induced cell migration.
**Fig. S10.** Immunofluorescence staining of lipid droplets.
**Table S1.** Oligonucleotide sequences for primers, probes and siRNAs.
**Table S2.** Antibodies used for western immunoblotting.

## Data Availability

The prostate cancer databases used in this study are available through cBioportal (https://www.cbioportal.org/): Prostate Adenocarcinoma (Fred Hutchinson CRC, *Nat Med* 2016); Metastatic Prostate Adenocarcinoma (MCTP, *Nature* 2012); Metastatic Prostate Cancer (SU2C/PCF Dream Team, *Cell* 2015); Prostate Adenocarcinoma (TCGA, PanCancer Atlas); Prostate Adenocarcinoma (TCGA, *Cell* 2015); Prostate Adenocarcinoma (MSKCC, *Cancer Cell* 2010); and Prostate Adenocarcinoma (SMMU, *Eur Urol* 2017).
