# Supplementary material for: The FABP12/PPARγ pathway promotes metastatic transformation by inducing epithelial‐to‐mesenchymal transition and lipid‐derived energy production in prostate cancer cells
Source: Mol Oncol. 2020 Oct 23;14(12):3100–20. doi: 10.1002/1878-0261.12818 (PMC7718947; doi:10.1002/1878-0261.12818)
Supplement: Supplementary file 1 — Fig. S1. Association of increased FABP12 gene copy numbers and mRNA levels with PCa metastasis and Gleason scores. Fig. S2. RT‐PCR analysis of FABP transcripts in prostate cancer cell lines. Fig. S3. FABP12 promotes cell migration in DU145. Fig. S4. Effect of transient and stable expression of FABP12 on PC3 cell proliferation. Fig. S5. Effects of FABP12 overexpression on cell morphology, growth and PPAR activation. Fig. S6. Immunohistochemical staining of EMT markers in PC3‐derived xenograft primary and metastatic tumor tissues. Fig. S7. FABP12‐induced cell motility in PC3 cells is reduced upon SNAI2 (Slug) depletion. Fig. S8. Effect of transient expression of FABP12 on PPARγ transactivation in PC3 cells. Fig. S9. PPARγ depletion attenuates FABP12‐induced cell migration. Fig. S10. Immunofluorescence staining of lipid droplets. Table S1. Oligonucleotide sequences for primers, probes and siRNAs. Table S2. Antibodies used for western immunoblotting. [file MOL2-14-3100-s001.pdf]

Supplementary Table S1. Oligonucleotide sequences for primers, probes and siRNAs

**Gene**	**Type**	**Strand**	**Sequence**	**Note**
FABP4	Primer	Sense	5'-GCAGCTTCCTTCTCACCTTG-3'	From IDT
		Antisense	5'-CATTCCACCACCAGTTTATC-3'	IDT
FABP5	Primer	Sense	5'-GAATACATGAAGGAGCTAGG-3'	IDT
		Antisense	5'-ACTGAGCTTGGTCATTCTC-3'	IDT
FABP9	Primer	Sense	5’-ATGGTTGAGCCCTTCTTGG-3’	IDT
		Antisense	5’-CACACCTTTTCGTAGATTCTG-3’	IDT
FABP12	Primer	Sense	5'-TCTGAGACAGAATGATTGACC-3'	IDT
		Antisense	5’-GTCAGTAGTTTGGATGACAGC-3’	IDT
CDH1	Primer	Sense	5’-ATTCAAAGTGGGCACAGATGG-3’	IDT
		Antisense	5’-AACATTGATTGCGTCACTCAG-3’	IDT
PPARγ	siRNA1	Sense	5’-CCAGUGGUUGCAGAUUACAAGUAUG-3’	Invitrogen
		Antisense	5’-CAUACUUGUAAUCUGCAACCACUGG-3’	Invitrogen
	siRNA2	Sense	5’-AGGGAGUUUCUAAAGAGCCUGCGAA-3’	Invitrogen
		Antisense	5’-UUCGCAGGCUCUUUAGAAACUCCCU-3’	Invitrogen
PPRE	Probe	Sense	5’-ACGAGTTCTAGGACAAAGGTCATGCA-3’	Consensus underlined
		Antisense	5’-GGCTGCATGACCTTTGTCCTAGAA-3’	

Supplementary Table S2. Antibodies used for Western immunoblotting

**Antibody**	**Company**	**Concentration**	**Secondary**
FABP12	Customized from BIOMATIC	1:350	Rabbit (1:10,000)
Slug	Cell Signaling Technology	1:1000	Rabbit (1:20,000)
E-Cadherin	Cell Signaling Technology	1:1000	Rabbit (1:20,000)
Vimentin	Cell Signaling Technology	1:1000	Rabbit (1:20,000)
β-Catenin	Cell Signaling Technology	1:1000	Rabbit (1:25,000)
HA	Roche	1:2000	Rat (1:25,000)
Lamin A/C	ThermoFisher	1:1000	Mouse (1:25,000)
ADRP	Cell Signaling Technology	1:1000	Rabbit (1:20,000)
FASN	Cell Signaling Technology	1:1000	Rabbit (1:20,000)
ACLY	Cell Signaling Technology	1:1000	Rabbit (1:20,000)
CPT1A	Cell Signaling Technology	1:1000	Rabbit (1:20,000)
α-Tubulin	DSHB	1:100,000	Mouse (1:25,000)
β-Actin	Sigma-Aldrich	1:100,000	Mouse (1:25,000)

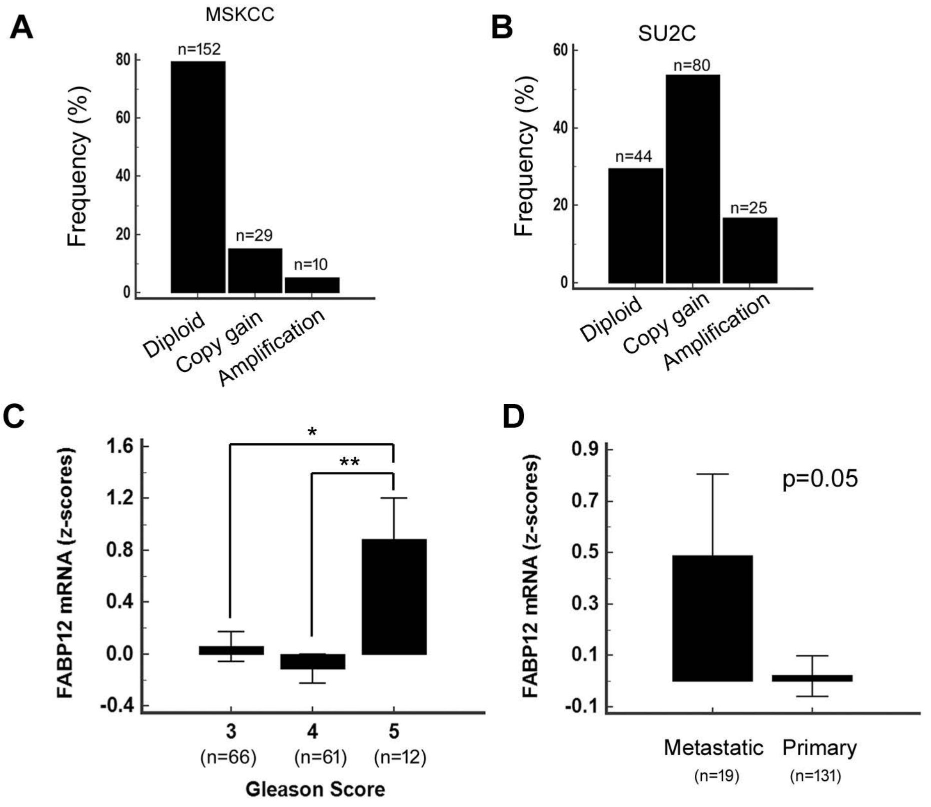

Figure S1. Association of increased *FABP12* gene copy numbers and mRNA levels with PCa metastasis and Gleason scores. (A, B) Increased relative frequency of tumors with *FABP12* gene copy gain and amplification in a metastatic PCa patient cohort (SU2C) compared to a primary PCa population (MSKCC). **(C, D)** *FABP12* mRNA levels are significantly associated with high Gleason scores (C) and metastasis (D) in a human PCa patient population. Gene copy number data were obtained through cBioportal (www.cbioportal.org). Statistical analysis: one way ANOVA (C) and student t-test (D). n, sample size; *, $p<0.05$; **, $p<0.01$.

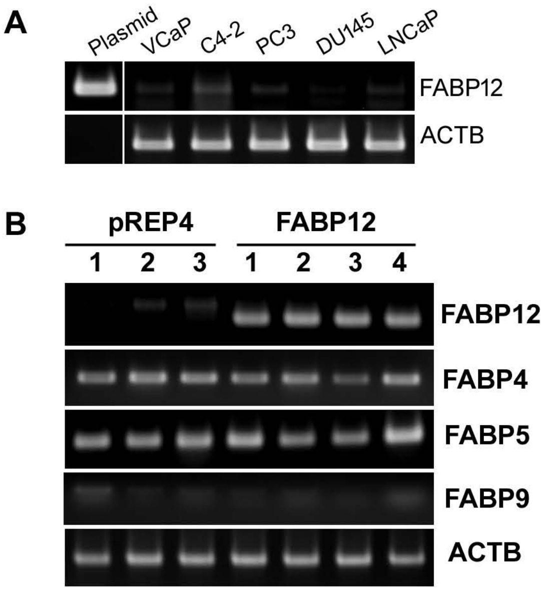

Figure S2. RT-PCR analysis of *FABP* transcripts in prostate cancer cell lines. Total RNA was isolated from PCa cell lines using Trizol reagent following the manufacturer's directions. (**A**) PCR products of FABP12 were generated by 35 cycles of PCR amplification with denaturation (30 seconds at 94°C), annealing (30 seconds at 55°C) and extension (1 minute at 72°C). pcDNA3.1 plasmid (Plasmid) containing the entire coding sequence of human *FABP12* cDNA (1 ng/mL) served as a positive control. (**B**) PCR products were generated from PC3 cells with stable transfection of empty plasmid (pREP4) or pREP4-FABP12 expression construct (FABP12) using primers targeting FABP4 (25 cycles), FABP5 (25 cycles), FABP9 (35 cycles) and FABP12 (35 cycles). Human β-actin (*ACTB*) served as control for cDNA template input. All cell lines were obtained from ATCC, with the exception of C4-2 which was a gift from Dr. Martin Gleave and LNCaP which was a gift from Dr. John Lewis.

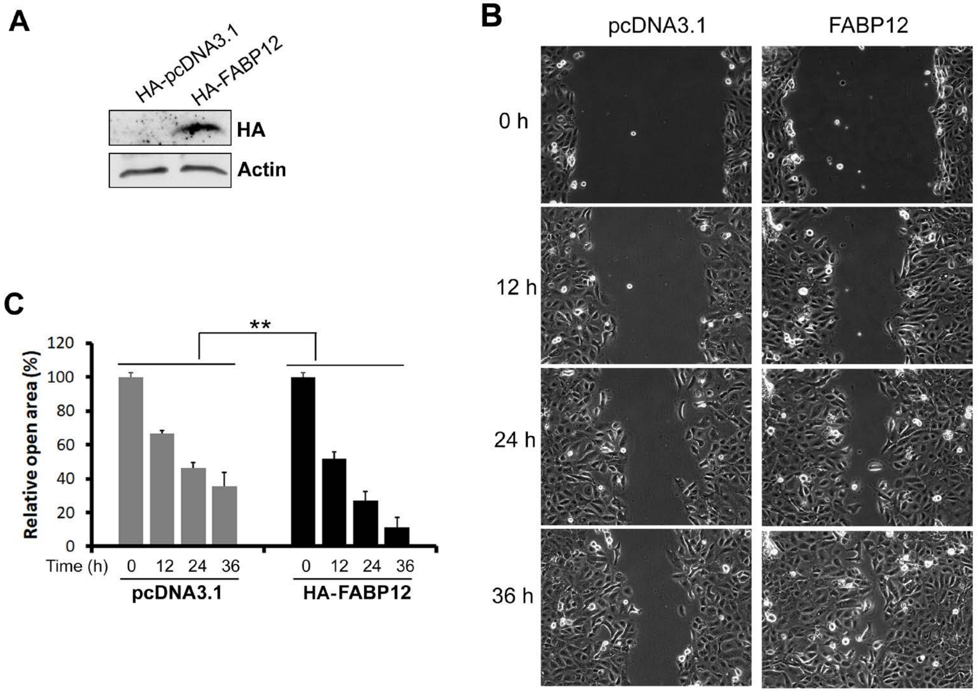

Figure S3. FABP12 promotes cell migration in DU145. Human prostate cancer cell line DU145 was transfected with either an empty pcDNA3.1 plasmid (HA-pcDNA3.1) or a FABP12 expression construct (HA-FABP12). (A) Western blot analysis confirms the expression of HA-tagged FABP12 in DU145 cells. (B) Representative image showing increased cell migration in FABP12-expressing DU145 cells using the scratch assay. (C) Histogram comparing relative open area (inverse indication of cell migration rate) between the control and FABP12-transfected DU145 cells at different time points as indicated. Data at each time point was collected from triplicate wells at two seperate positions (n=6). Significance of difference was analyzed by two-way ANOVA. “**” denotes $p<0.01$; h, hours.

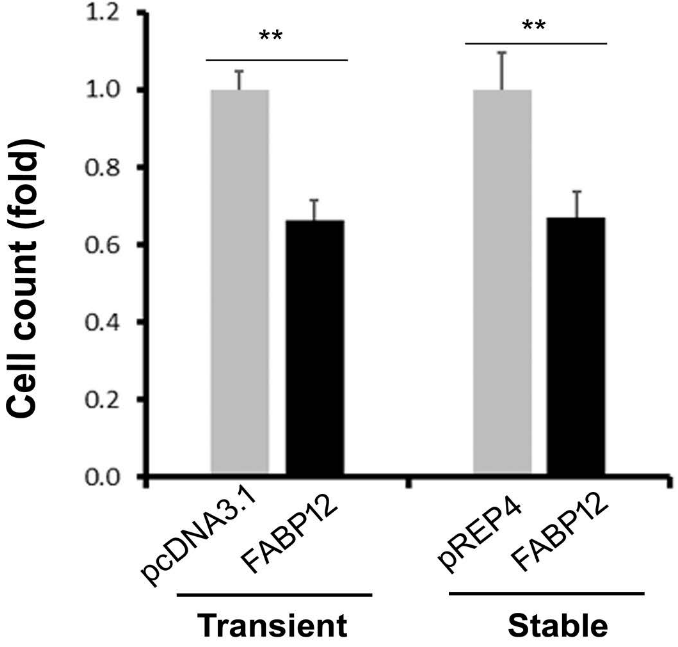

Figure S4. Effect of transient and stable expression of FABP12 on PC3 cell proliferation. Cells were seeded at 30,000 cells/well and cultured in 12-well plates in triplicate (n=3) for three days. Cells were counted using a Coulter Particle and Size Analyzer. Numbers of FABP12-expressing PC3 cells (FABP12) are relative to their corresponding control cells (pcDNA3.1 or pREP4) normalized to 1. The student t-test was used for statistical analysis. ** denotes $p<0.01$.

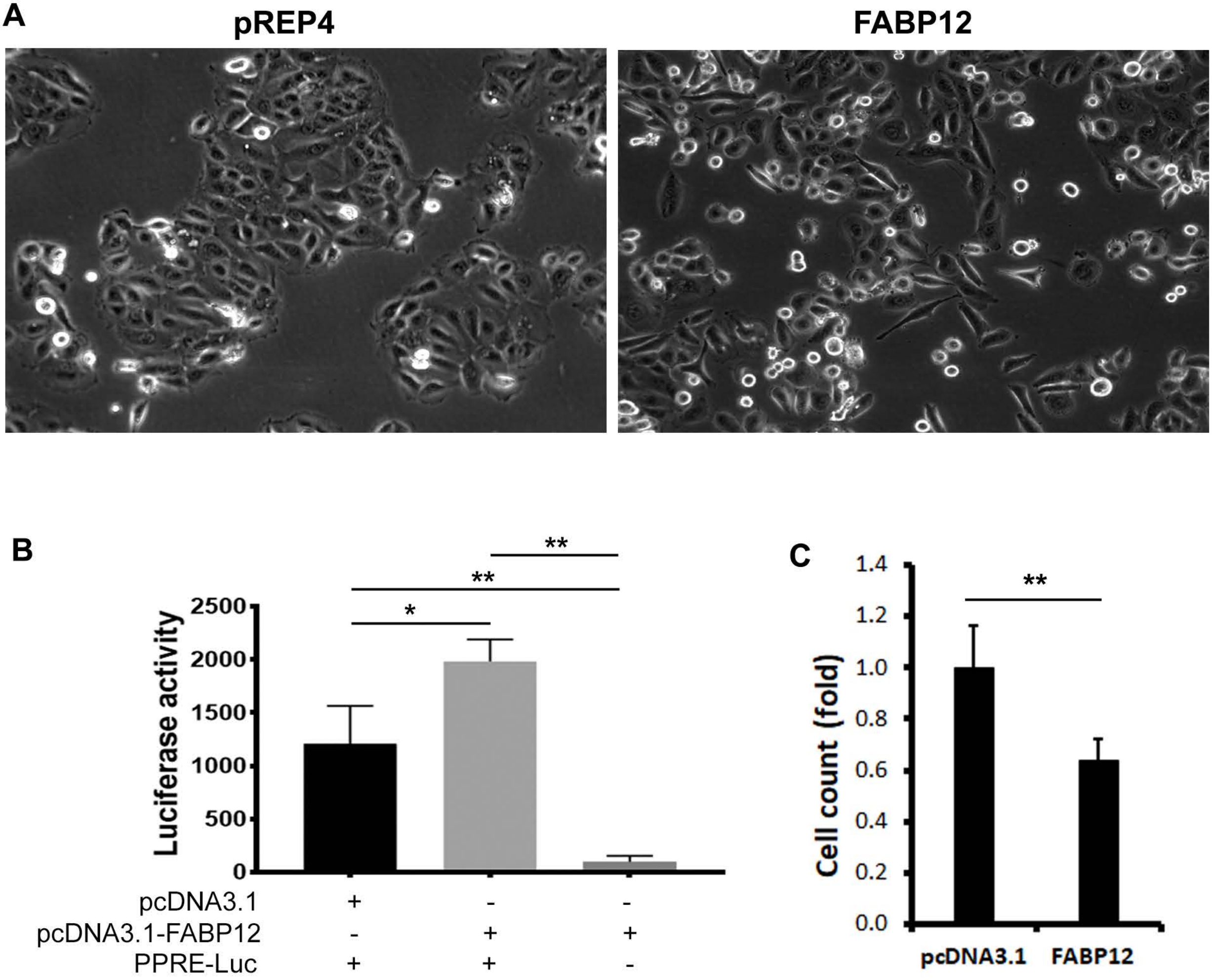

Figure S5. Effects of FABP12 overexpression on cell morphology, growth and PPAR activation. (A) DU145 cells with stable ectopic expression of FABP12 are elongated and enlarged compared to pREP4 control cells. (B) Transienty-transfected DU145-FABP12 cells show significantly increased PPRE-driven luciferase compared to pcDNA3.1 control cells, indicative of PPAR activation. (C) Transiently-transfected DU145-FABP12 show significantly reduced growth rates compared to pcDNA3.1 control cells. Statistical analysis: one way ANOVA (B) and student t-test (C). * denotes $p<0.05$; **, $p<0.01$.

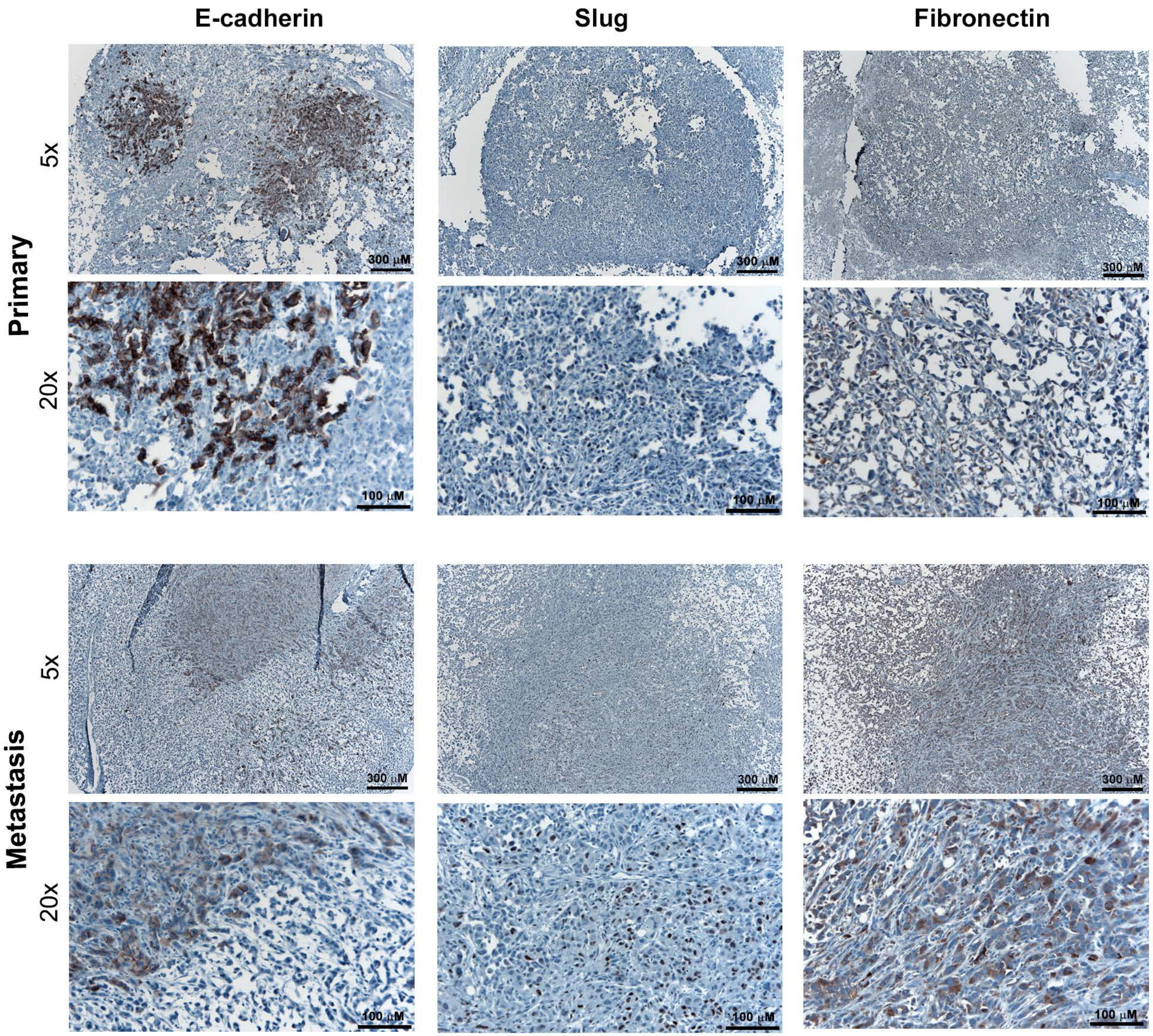

Figure S6. Immunohistochemical staining of EMT markers in PC3-derived xenograft primary and metastatic tumor tissues. Compared to primary tumor tissues, metastatic tumors showed decreased E-cadherin, and increased Slug and fibronectin protein levels.

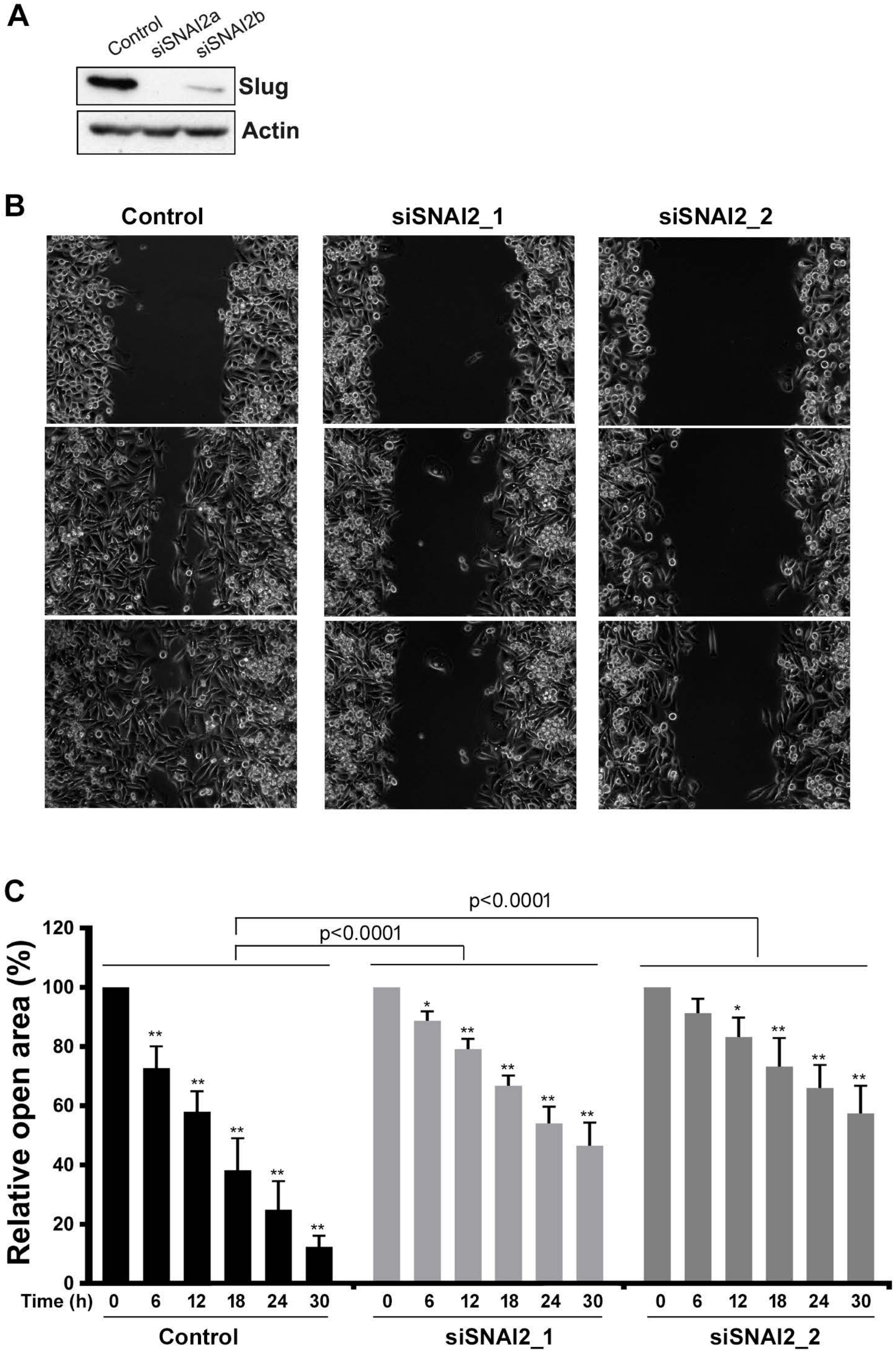

Figure S7. FABP12-induced cell motility in PC3 cells is dependent on SNAI2 (Slug). (A) Western blot showing depletion of Slug in PC3-pREP4-FABP12 cells transfected with siRNAs targeting SNAI2 (siSNAI2_1, siSNAI2_2). **(B, C)** Scratch assay images (B) and statistical bar charts (C) showing reduced cell motility in PC3-pREP4-FABP12 cells upon SNAI2 depletion. Statistical analysis: two-way ANOVA. * denotes $p<0.05$, **, $p<0.01$.

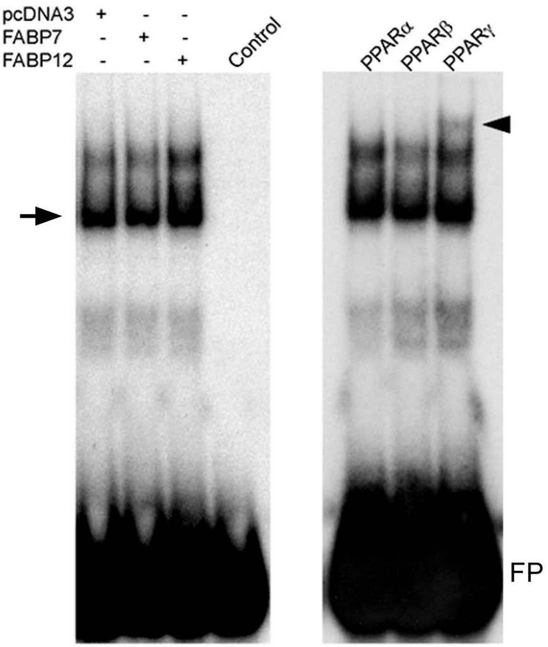

Figure S8. Effect of transient expression of FABP12 on PPARγ transactivation in PC3 cells. Left panel: Gel shift assay with nuclear lysates from PCa cell line PC3 transfected with empty pcDNA3, pcDNA3-FABP7 (FABP7 is not expressed in PCa cells) and pcDNA3-FABP12 expression constructs. Peroxisome proliferator response element (PPRE) oligonucleotides were annealed and radiolabelled with α-^{32}P-dCTP. This probe was used to study PPRE-PPAR interaction (indicative of PPAR activation). The arrow points to protein-PPRE complexes in the indicated PC3 nuclear lysates. FABP12 overexpression enhances PPAR activation in PC3 cells. Right panel: Supershift assay using antibodies against PPARα, β and γ suggest specific interaction of PPRE with PPARγ in FABP12-overexpressing PC3 cells (arrowhead). FP, free probe.

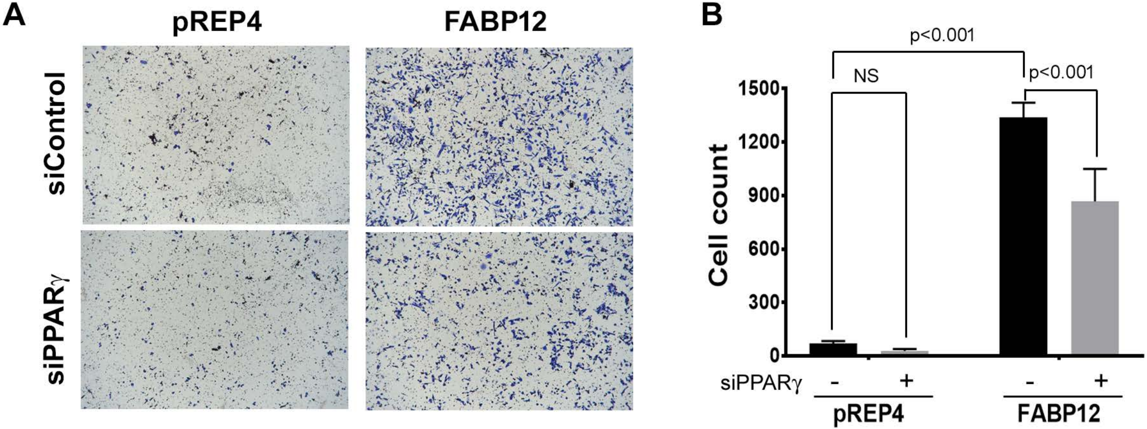

Figure S9. PPARγ depletion attenuates FABP12-induced cell migration. (A) PC3 control (pREP4) and FABP12-expressing (FABP12) stable transfectants were transfected with control (siControl) and PPARγ-specific siRNA (siPPARγ). Cell migration was then analyzed with Transwell inserts (BD Biosciences) following the manufacturer's protocol. **(B)** Histogram comparing cell numbers on the resulting Transwell membranes. Experiments were repeated three times. The two-sided student t-test was used for statistical analysis. NS denotes not significant.

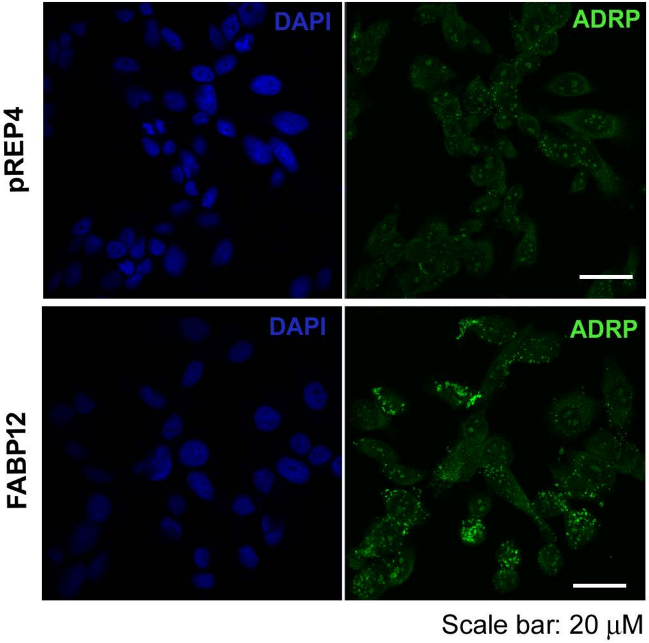

Figure S10. Immunofluorescence staining of lipid droplets. PC3 stable cell lines transfected with empty vector (pREP4) or pREP4-FABP12 expression construct (FABP12) were immunostained with anti-ADRP (a lipid droplet marker) antibody at a dilution of 1:50. The signal was visualized with Alexa-488-conjugated goat anti-rabbit IgG. Images show enhanced ADRP immunoreactivity in FABP12-expressing PC3 cells.
